# Effect of Salt Stress on Growth, Physiological Parameters, and Ionic Concentration of Water Dropwort (*Oenanthe javanica*) Cultivars

**DOI:** 10.3389/fpls.2021.660409

**Published:** 2021-06-21

**Authors:** Sunjeet Kumar, Gaojie Li, Jingjing Yang, Xinfang Huang, Qun Ji, Zhengwei Liu, Weidong Ke, Hongwei Hou

**Affiliations:** ^1^The State Key Laboratory of Freshwater Ecology and Biotechnology, The Key Laboratory of Aquatic Biodiversity and Conservation of Chinese Academy of Sciences, Institute of Hydrobiology, Chinese Academy of Sciences, Wuhan, China; ^2^University of Chinese Academy of Sciences, Beijing, China; ^3^Institute of Vegetables, Wuhan Academy of Agricultural Sciences, Wuhan, China

**Keywords:** antioxidants, reactive oxygen species, ions, growth, NaCl, water dropwort

## Abstract

Salt stress is an important environmental limiting factor. Water dropwort (*Oenanthe javanica*) is an important vegetable in East Asia; however, its phenotypic and physiological response is poorly explored. For this purpose, 48 cultivars of water dropwort were grown hydroponically and treated with 0, 50, 100, and 200 mm NaCl for 14 days. Than their phenotypic responses were evaluated, afterward, physiological studies were carried out in selected sensitive and tolerant cultivars. In the present study, the potential tolerant (V11E0022) and sensitive (V11E0135) cultivars were selected by screening 48 cultivars based on their phenotype under four different levels of salt concentrations (0, 50, 100, and 200 mm). The results depicted that plant height, number of branches and leaves were less effected in V11E0022, and most severe reduction was observed in V11E0135 in comparison with others. Than the changes in biomass, ion contents, accumulation of reactive oxygen species, and activities of antioxidant enzymes and non-enzymatic antioxidants were determined in the leaves and roots of the selected cultivars. The potential tolerant cultivar (V11E0022) showed less reduction of water content and demonstrated low levels of Na^+^ uptake, malondialdehyde, and hydrogen peroxide (H_2_O_2_) in both leaves and roots. Moreover, the tolerant cultivar (V11E0022) showed high antioxidant activities of ascorbate peroxidase (APX), superoxide dismutase, peroxidase, catalase (CAT), reduced glutathione (GSH), and high accumulation of proline and soluble sugars compared to the sensitive cultivar (V11E0135). These results suggest the potential tolerance of V11E0022 cultivar against salt stress with low detrimental effects and a good antioxidant defense system. The observations also suggest good antioxidant capacity of water dropwort against salt stress. The findings of the present study also suggest that the number of branches and leaves, GSH, proline, soluble sugars, APX, and CAT could serve as the efficient markers for understanding the defense mechanisms of water dropwort under the conditions of salt stress.

## Introduction

Salinity is one of the major abiotic stresses that has been significantly affecting the plant growth and yield (Gharsallah et al., 2016). The continuous increase in salinity in arable land due to poor cultivation practices and climate change have devastating global effects, and it is estimated that about 50% of arable land will be lost by the middle of the 21st century (Islam et al., 2019). To date, about 1,125 million hectares of agricultural lands have already been seriously affected by salinity, thus it is considered a serious threat to agriculture (Islam et al., 2019; Sanower-Hossain, 2019). In China, a total of 36.7 million hectares of land has been greatly affected by salinity, of which 12.3 million hectares is agricultural land (Li-ping et al., 2015).

A high level of salt results ionic imbalance and osmotic stress in plants which causes severe effects on morphology, biomass, and biochemical processes of the plants, and ultimately result in plant damages (Zhang et al., 2013; Rahneshan et al., 2018). Soil salinity enhances the Na^+^ and Cl^−^ contents in plants which then increases the ratio of Na^+^/K^+^, which ultimately affects the regular ionic activities in plants (Singh et al., 2014). Several plants have developed different strategies to overcome these challenges. Among these, the first one is the maintenance of homeostasis by the osmotic adjustment that carries out the excessive Na^+^ ions to the vacuole, and the second is the synthesis of osmolyte to cope with this situation (Queirós et al., 2009; Silva et al., 2015; Rahneshan et al., 2018). A high K^+^/Na^+^ ratio also plays a vital role in maintaining membrane potential as well as osmotic and turgor pressures. It also helps in enzyme activation and tropisms (Rahneshan et al., 2018). Plants produce osmolytes, such as proline and soluble sugars protect the plant cells against the adverse effects of salt stress. These help in osmotic adjustment, and their higher production can increase the salinity tolerance (Rahneshan et al., 2018). Similarly, antioxidant molecules including glutathione (GSH) and proteins have the role in control of concentration of reactive oxygen species (ROS), which ultimately help in salinity tolerance. Proteins can also help in osmotic adjustment under salt stress (Zhang et al., 2013; Hasanuzzaman et al., 2018).

Salt stress also leads to increasing the level of ROS which results in oxidative stress, which in turn affects the plants both at cellular and metabolic levels (Ali et al., 2017; Sahin et al., 2018). The plants overcome the oxidative damage through activation of antioxidants through enzymatic and non-enzymatic mechanisms. The enzymatic component includes superoxide dismutase (SOD; EC 1.15.1.1), peroxidase (POD; EC 1.11.1.7), catalase (CAT; EC 1.11.1.6), and ascorbate peroxidase (APX; EC 1.11.1.1; Shaheen et al., 2013; Shafeiee and Ehsanzadeh, 2019; Soares et al., 2019; Sarker and Oba, 2020b). Moreover, the ROS, such as superoxide radicals ($O_{2}^{-}$), hydrogen peroxide (H_2_O_2_), and small amounts of transition metals, also increases the concentration of OH^−^. Therefore, plants carry out detoxification to avoid the oxidative damage where these antioxidant enzymes play an important role. A study reported that the antioxidant enzymes positively correlate with the plant tolerance in drought and salt stress (Wang et al., 2009). Moreover, the higher antioxidant activities can help improving death in plants (Khan et al., 2017).

*Oenanthe javanica* (Blume) DC (also known as water dropwort) is an aquatic perennial herb belonging to the family Apiaceae. It is mainly cultivated in East Asian countries, such as China, Japan, Korea, Thailand, Malaysia, and Australia (Jeon et al., 2007; Lee and Kim, 2009; Lu and Li, 2019). Water dropwort contains high contents of minerals and vitamins, and demonstrates medicinal properties (Jiang et al., 2015; Lu and Li, 2019; Kumar et al., 2020, 2021). It has been traditionally used as a vegetable in China. Various researchers have suggested that persicarin, isorhamnetin, and hyperoside are the three important compounds present in *O. javanica*, which possess the pharmacological activities for curing various ailments (Jiang et al., 2015; Chan et al., 2017; Lu and Li, 2019). Therefore, all these properties make the water dropwort a popular edible plant in China. The previous studies reported that *O. javanica* is sensitive to drought and salt stress, and these are the key limiting factors for its growth and production (Jiang et al., 2015; Kumar et al., 2020). There is only limited information available related to the salt tolerance mechanism of water dropwort concerning the regulation of free radicals quenching pathway with the antioxidative defense.

The present study is designed to access phenotypic responses of different water dropwort cultivars under salt stress and to select salt-tolerant and sensitive cultivars based on phenotype among them. Secondly, it aims to study some physiological parameters including enzymatic and the non-enzymatic antioxidant defense system, chlorophyll content, and ionic homeostasis regarding the salt tolerance in selected tolerant and sensitive cultivars of water dropwort. For these objectives, various parameters, such as plant growth, fresh and dry biomass, relative water content (RWC), chlorophyll content, Na^+^ and K^+^ content, production rate of ROS, osmolytes and antioxidant molecules concentration, and activities of antioxidant enzymes, were studied.

## Materials and Methods

### Plant Culture and Salt Treatment

Seeds of 48 cultivars of *Oenanthe javanica* were kept in wet sand for 1 month and then shifted to the wet filter paper and placed in the growth chamber (12/12 h) at 25°C. After germination for 7–10 days, seeds were transferred to Hoagland nutrient solution (Hoagland and Arnon, 1950) and grown for 44 days in greenhouse condition at 20–25°C for 16 h photoperiod. The composition of media was 3.59 mm Ca(NO_3_)_2_, 8.7 mm KNO_3_, 0.713 mm N₂H₄O₃, 1.516 mm MgSO_4_, 1.314 mm KH_2_PO_4_, 62.5 μm FeSO_4_, 44.6 μm EDTA, 48.5 μm H_3_BO_3_, 13.2 μm MnSO_4_, 1.36 μm ZnSO_4_, 0.501 μm CuSO_4_, and 2.55 μm (NH_4_)_2_MoO_4_. Initially, the plants were grown hydroponically for 30 days than these plants were treated with 0 (control), 50, 100, and 200 mm NaCl for 14 days. Afterward, these treated plants were used for further analysis. All experiments were conducted in biological triplicates.

### Morphological Parameters and Chlorophyll Content

After harvesting, morphological parameters, such as plant height, stem length, root length, and number of branches and leaves, were measured. The total chlorophyll content was determined using the SPAD-502Plus (Konica Minolta, Japan). The fresh and dry biomass of selected cultivars was also measured. The shoots biomass and roots biomass were determined after washing with distilled water and drying them gently on a paper towel. The dry weight (DW) was determined after drying for 72 h at 70°C.

### Determination of RWC

Relative water content (RWC) of leaves was measured according to the method described by Sarker and Oba (2018) and Kumar et al. (2020). After determining the fresh weight (FW), leaves were immersed in distilled water in a closed Petri dish for 4 h, and the turgor weight (TW) of each leaf was noted. Thereafter, the leaf samples were placed in a pre-heated oven at 70°C for 24 h to obtain dry weight (DW). Afterward, RWC was calculated using the following formula:

$$\mathrm{RWC}\;\left(\%\right)\;=\frac{\left(\mathrm{FW}-\mathrm{DW}\right)}{\left(\mathrm{TW}-\mathrm{DW}\right)}\;\times \;100$$

### Determination of Na^+^ and K^+^ Contents

For determination of Na^+^ and K^+^ contents, approximately 100 mg of dried leaves and roots was digested with 6 ml nitric acid in a microwave digestion system (Multiwave 3000, Anton Paar, Austria) for 90 min. The digested samples were diluted up to 10 ml with ultra-deionized water. The ions concentrations were determined by using the inductively coupled plasma-atomic emission spectroscopy ICP-OES (Optima8000, PerkinElmer, United States; Colomer-Winter et al., 2018).

### Determination of Photosynthetic Pigments

For determination of chlorophyll and carotenoid concentrations, approximately 100 mg of fresh leaves was homogenized with 80% acetone and centrifuged at 7,000 × *g* for 10 min. The supernatant was collected, and the absorbance (A) was measured at 663 nm for chlorophyll *a*, 646 nm for chlorophyll *b*, and 470 nm for carotenoid using an ELISA plate reader (i3× molecular devices, United States; Sarker and Oba, 2020a; Kumar et al., 2021). The concentration of chlorophyll and carotenoids was calculated as follows:

$$\mathrm{Chlorophyll}\;a\;=\;12.21\left(A663\right)-2.81\left(A646\right)$$

$$\mathrm{Chlorophyll}\;b\;=\;20.13\left(A646\right)-5.03\left(A663\right)$$

$$\mathrm{Total chlorophyll}\;\left(a+b\right)\;=\;\mathrm{chl}\;a+\mathrm{chl}\;b$$

$$\mathrm{Carotenoids}\;=\;\left[1000\left(A470\right)-3.27\left(\mathrm{chl}\;a\right)-104\left(\mathrm{chl}\;b\right)\right]/229$$

### Determination of Lipid Peroxidation

For determination of malondialdehyde (MDA), approximately 50 mg of fresh leaves and roots was homogenized with 450 μl phosphate buffer saline (PBS; pH 7.4, 0.1 M) with a glass homogenizer. The samples were then centrifuged three times at 4,000 × *g* for 15 s with intervals of 30 s. Afterward, the homogenate was centrifuged at 3500 × *g* for 10 min. After centrifugation, the supernatant was used for the MDA analysis with a commercially available test kit (A003-1-1, Nanjing Jiancheng Bioengineering Institute, Nanjing, China). Finally, absorbance was measured at 530 nm (Dai et al., 2018).

### Assays for Hydrogen Peroxide, Proteins, GSH, and Antioxidant Enzymes

For determination of H_2_O_2_, GSH, and antioxidant enzymes, approximately 200 mg of fresh leaves was homogenized with 1.8 ml of PBS (pH 7.4, 0.1 M) with a glass homogenizer and then centrifuged at 3,500 × *g* for 12 min. The supernatant was used for determination of total protein, H_2_O_2_, GSH contents, and antioxidant enzymes activities including APX, SOD, POD, and CAT with commercially available test kits (Nanjing Jiancheng Bioengineering Institute, Nanjing, China; Zhang et al., 2015; Hussain et al., 2016; Dai et al., 2018; Yang et al., 2018).

The Coomassie brilliant blue method was used for determining the total protein content with a commercially available total protein assay kit (A045-2; Nanjing Jiancheng Bioengineering Institute, China), and the absorbance was measured at 595 nm. H_2_O_2_ forms a complex with molybdate whose absorbance was measured at 405 nm. The GSH content was determined with a glutathione assay kit (A006-1; Nanjing Jiancheng Bioengineering Institute, China) according to the DTNB [5,5,-dithiobis (2-nitrobenzoic acid)] method. The absorbance was measured at 420 nm, and GSH content was expressed as mg g^−1^ protein (Zhang et al., 2015; Hussain et al., 2016; Dai et al., 2018; Yang et al., 2018).

The activity of APX was determined with the APX assay kit (A123-1-1; Nanjing Jiancheng Bioengineering Institute, China). APX catalyzed the oxidation of ascorbate at 290 nm and expressed as U mg^−1^ FW. One unit activity of APX is the amount of enzyme, which oxidizes 1 μmol ascorbate per min in 1 mg fresh sample (Nakano and Asada, 1981). The activity of SOD was determined with SOD assay kit (A001-1; Nanjing Jiancheng Bioengineering Institute, China) and was presented as U mg^−1^ FW. One unit of SOD activity is the amount of extract that gives 50% inhibition in reducing xanthine monitored at 550 nm (McCord and Fridovich, 1969). The activity of POD was measured by using a POD assay kit (A084-3-1; Nanjing Jiancheng Bioengineering Institute, China) on the basis of guaiacol oxidation at 470 nm by H_2_O_2_ and expressed as U mg^−1^. The change in absorbance at 470 nm was recorded every 20 s (Chance and Maehly, 1955). One unit of POD activity is the amount of enzyme, which causes the decomposition of 1 μg substrate per minute in 1 mg fresh sample at 37°C. Similarly, the activity of CAT was measured with a CAT assay kit (A007-1; Nanjing Jiancheng Bioengineering Institute, China) and was presented as U mg^−1^ FW. One unit of CAT activity is the amount of enzyme which causes the decomposition of 1 μmol H_2_O_2_ per minute in 1 mg fresh sample at 37°C (Beers and Sizer, 1952).

### Determination of Concentrations of Proline and Soluble Sugars

Approximately 100 mg of fresh leaves and roots was homogenized for determination of proline content following the manufacturer’s instructions (A107-1-1, Nanjing Jiancheng Bioengineering Institute, Nanjing, China), and the absorbance was measured at 520 nm. For the analysis of soluble sugars, approximately 0.1 g of fresh samples was homogenized in 1 ml ddH_2_O with a glass homogenizer. The tubes were boiled at 95°C for 10 min and cooled with tap water. After cooling, the homogenate was centrifuged at 4,000 × *g* for 10 min. Thereafter, the supernatant was diluted with ddH_2_O at 1:9. The diluted extracts were used for determination of soluble sugar content using a commercially available test kit (A145-1-1, Nanjing Jiancheng Bioengineering Institute, Nanjing, China). Finally, the absorbance was measured at 620 nm and the soluble sugar concentration expressed and the results expressed in the fresh weight (FW) basis (Dai et al., 2018; Kumar et al., 2021).

### Statistical Analysis

All experiments were performed in triplicates, and SPSS 25.0 statistical program (IBM Crop. Armonk, NY, United States) was used for statistical analysis. Tukey tests were performed for determining the significant differences (*p* ≤ 0.05) among treatments. GraphPad Prism 7 (San Diego, California, United States) was used for figures, and significant differences were indicated by different letters. All data are presented as mean ± standard error (SE).

## Results

### Growth and Biomass of Water Dropwort

The growth properties of 48 cultivars were strongly influenced by the salt stress. The phenotypic parameters of water dropwort, such as plant height, stem length, root length, number of branches, and number of leaves in all treatments, were significantly lower than the control (*p* < 0.05; Supplementary Table S1). The plant growth showed an inverse relation with the different level of salt stress imposed. Moreover, a high reduction in the plant height, root length, stem length, and number of branches, and leaves was observed in all cultivars at 200 NaCl (Supplementary Table S1). Beside the plant height, the drastic effects of salinity were found in the number of branches and leaves of all cultivars. Furthermore, an increase in salt concentration caused a decline in the number of branches and leaves. Based on the phenotypic results, we identified V11E0022 as the potential tolerant cultivar, whereas the V11E0135 as the most sensitive cultivar among the 48 cultivars under consideration in the present study (Supplementary Table S1).

**Figure 1:**
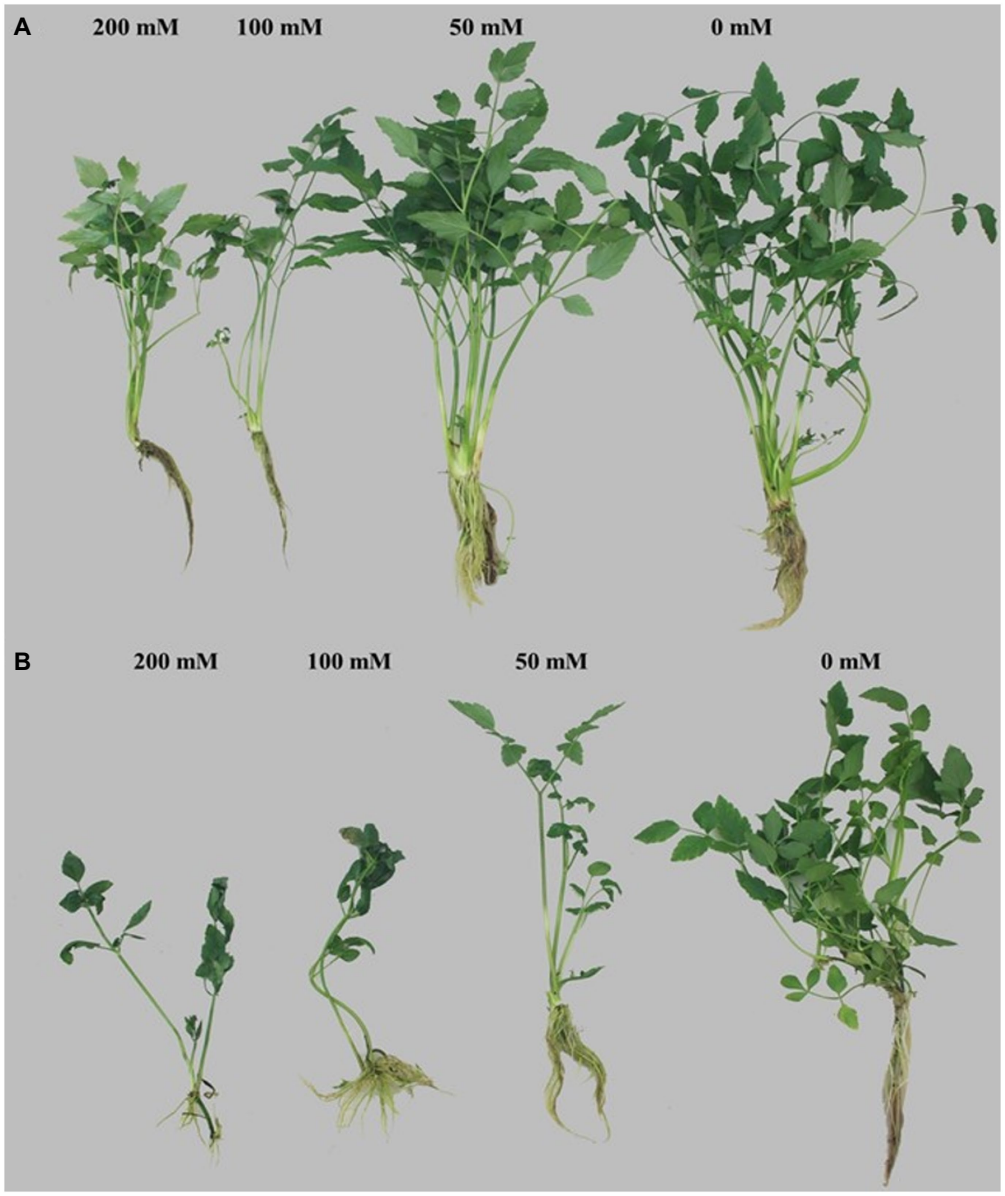
Effect of salt stress on the tolerant and sensitive cultivars of water dropwort. **(A)** potential tolerant cultivar (V11E0022) and **(B)** sensitive cultivar (V11E0135).

The growth parameters of selected tolerant and sensitive cultivars were greatly affected by the salt stress (Figure 1). A gradual decrease in the plant height, and root and stem length of both cultivars were observed under all treatments (50, 100, and 200 mm) in comparison with the control, and a maximum reduction was detected at 200 mm NaCl (Table 1). The plant height of V11E0135 was decreased by 28.5 and 31.4% by exposure of 100 and 200 mm NaCl, respectively, while it decreases only 16.5 and 22.7% in V11E0022 under 100 and 200 mm NaCl, respectively. Similarly, the number of branches and leaves of both cultivars were significantly reduced under different levels of NaCl compared to the control (*p* < 0.05). The number of branches in V11E0135 was reduced by 68.5 and 76.7% by exposure of 100 and 200 mm NaCl, respectively, whereas the reduction in V11E0022 was only 33.7 and 37.5% under 100 and 200 mm NaCl, respectively. Similarly, the number of leaves of V11E0135 decreased by 74.5 and 84.7% under 100 and 200 mm NaCl, respectively. However, V11E0022 showed only 31.3 and 34.9% reduction in number of leaves under 100 and 200 mm NaCl, respectively. The salt stress also significantly reduced the fresh and dry weight of the shoot and root (*p* < 0.05), and a maximum reduction was observed under 200 mm NaCl in V11E0135 when compared with the control (Table 2). The shoot fresh weight of V11E0135 was decreased by 70% at 100 mm and 80.3% at 200 mm NaCl. On the other hand, V11E0022 showed 40.7 and 45.3% decrease in shoot fresh weight under 100 and 200 mm NaCl, respectively. Furthermore, root fresh weight of V11E0135 decreased by 61.9 and 71.63% under 100 and 200 mm NaCl, respectively. In contrast, V11E0022 showed reduction of 47.5 and 51.1% at 100 and 200 mm NaCl, respectively. Overall, the shoot and root (fresh and dry) weight of the V11E0135 cultivar was reduced more than that of V11E0022. Overall, V11E0135 showed drastic effects for different growth parameters compared to the V11E0022 cultivar (Table 1).

**Table 1:** Effect of salt stress on morphological parameters and relative water content (RWC) of two cultivars of water dropwort.

Cultivars	NaCl (mm)	Plant height (cm)	Root length (cm)	Stem length (cm)	Number of branches	Number of leaves	RWC (%)
V11E0022	0	68.3 ± 1.5^c^	23.0 ± 1.0^c^	45.3 ± 0.6^c^	8.0 ± 0.0^c^	63.0 ± 3.0^c^	86.1 ± 4.2^bc^
	50	61.3 ± 1.5^b^	21.0 ± 1.0^bc^	40.3 ± 1.2^b^	6.3 ± 0.6^b^	51.3 ± 1.5^b^	93.0 ± 3.8^c^
	100	57.0 ± 2.0^ab^	19.3 ± 0.6^b^	37.7 ± 1.5^ab^	5.3 ± 0.6^ab^	43.3 ± 1.5^a^	85.9 ± 2.3^b^
	200	52.8 ± 1.3^a^	16.5 ± 0.5^a^	36.3 ± 1.5^a^	5.0 ± 0.0^a^	41.0 ± 1.7^a^	75.4 ± 3.4^a^
V11E0135	0	58.3 ± 1.5^c^	21.3 ± 0.6^c^	37.0 ± 1.0^b^	7.3 ± 0.6^c^	64.3 ± 1.5^d^	88.9 ± 4.6^b^
	50	46.3 ± 1.2^b^	19.0 ± 0.5^b^	27.3 ± 0.8^a^	3.7 ± 0.6^b^	27.3 ± 0.6^c^	94.3 ± 3.8^b^
	100	41.7 ± 1.5^a^	15.0 ± 0.5^a^	26.7 ± 0.3^a^	2.3 ± 0.6^ab^	16.3 ± 0.6^b^	81.2 ± 3.8^b^
	200	40.0 ± 1.5^a^	14.0 ± 0.5^a^	26.0 ± 1.0^a^	1.7 ± 0.6^a^	9.7 ± 1.5^a^	67.9 ± 2.4^a^

Different letters indicate a significant difference (*p* < 0.05) among the four treatments according to the Tukey test. The values are means ± SE.

**Table 2:** Effect of salt stress on fresh (FW) and dry weight (DW) of the shoot and root of two water dropwort cultivars.

	NaCl (mm)	V11E0022		V11E0135	
		FW	DW	FW	DW
Shoot biomass (mg plant^−1^)	0	50.93 ± 0.59^d^	3.69 ± 0.04^c^	46.51 ± 1.07^d^	3.58 ± 0.09^d^
	50	38.99 ± 1.72^c^	2.90 ± 0.14^b^	22.43 ± 0.80^c^	1.80 ± 0.09^c^
	100	30.19 ± 1.02^b^	2.34 ± 0.05^a^	13.93 ± 0.67^b^	1.14 ± 0.05^b^
	200	27.85 ± 0.06^a^	2.19 ± 0.01^a^	9.16 ± 0.36^a^	0.72 ± 0.03^a^
Root biomass (mg plant^−1^)	0	5.62 ± 0.35^b^	0.338 ± 0.017^b^	4.83 ± 0.31^c^	0.255 ±0.02^d^
	50	3.16 ± 0.19^a^	0.239 ±0.02^a^	2.95 ± 0.18^b^	0.187 ± 0.01^c^
	100	2.95 ± 0.19^a^	0.234 ± 0.02^a^	1.84 ± 0.13^a^	0.150 ± 0.01^b^
	200	2.75 ± 0.21^a^	0.225 ± 0.01^a^	1.37 ± 0.09^a^	0.108 ± 0.01^a^

Different letters indicate a significant difference (*p* < 0.05) among the four treatments according to the Tukey test. The values are means ± SE.

### Relative Water Content

Similarly, RWC decreased in both cultivars with the increase of NaCl compared to the control, except for 50 mm, where RWC was increased to 7.99 and 6.06% in V11E0022 and V11E0135, respectively (Table 1). Moreover, a relatively higher reduction of RWC was observed in V11E0135 compared to the V11E0022.

### Na^+^ and K^+^ Concentrations

The salt stress significantly enhanced the Na^+^ content in the roots and leaves of both cultivars under all treatments (50, 100, and 200; *p* < 0.05). Furthermore, the leaves and roots of V11E0135 showed high uptake of Na^+^ ion than its counterpart, and the highest Na^+^ content was detected under 200 mm NaCl (Table 3). Similarly, the K^+^ content in the roots and leaves of both cultivars decreased with increasing NaCl concentration, and the lowest level of K^+^ uptake was observed in V11E0135 at 200 mm NaCl. Moreover, a negative relationship was found between the salt stress and K^+^/Na^+^ ratio in both cultivars (Table 3).

**Table 3:** Changes in leaf and root ionic contents of two water dropwort cultivars under salt stress.

Cultivars	NaCl	Leaf ions (mg g^−1^ DW)			Root ions (mg g^−1^ DW)		
		Na^+^	K^+^	K^+^/Na^+^	Na^+^	K^+^	K^+^/Na^+^
V11E0022	0	3.27 ± 0.15^a^	70.93 ± 1.16^b^	21.70 ± 1.26^b^	1.05 ± 0.05^a^	54.05 ± 1.93^d^	51.50 ± 3.93^b^
	50	19.23 ± 0.17^b^	69.36 ± 0.84^b^	3.61 ± 0.04^a^	10.94 ± 0.52^b^	40.80 ± 1.00^c^	3.74 ± 0.25^a^
	100	28.13 ± 0.32^c^	69.59 ± 0.94^b^	2.44 ± 0.05^a^	14.01 ± 0.66^c^	23.19 ± 1.10^b^	1.66 ± 0.05^a^
	200	33.57 ± 0.44^d^	64.83 ± 1.04^a^	1.93 ± 0.03^a^	16.10 ± 0.78^d^	17.57 ± 0.67^a^	1.09 ± 0.05^a^
V11E0135	0	4.47 ± 0.10^a^	75.80 ± 0.96^c^	16.94 ± 0.23^c^	0.98 ± 0.04^a^	50.01 ± 2.23^c^	50.85 ± 2.05^c^
	50	27.57 ± 0.38^b^	74.54 ± 1.06^c^	2.71 ± 0.04^b^	13.33 ± 0.45^b^	47.32 ± 1.25^c^	3.55 ± 0.10^b^
	100	30.29 ± 0.46^c^	68.93 ± 1.07^b^	2.28 ± 0.02^b^	18.32 ± 0.80^c^	27.58 ± 0.79^b^	1.51 ± 0.10^a^
	200	45.27 ± 1.22^d^	63.59 ± 0.89^a^	1.40 ± 0.05^a^	26.16 ± 1.59^d^	14.76 ± 0.62^a^	0.57 ± 0.04^a^

Different letters indicate a significant difference (*p* < 0.05) among the four treatments according to the Tukey test. The values are means ± SE.

### Photosynthetic Pigments

**Figure 2:**
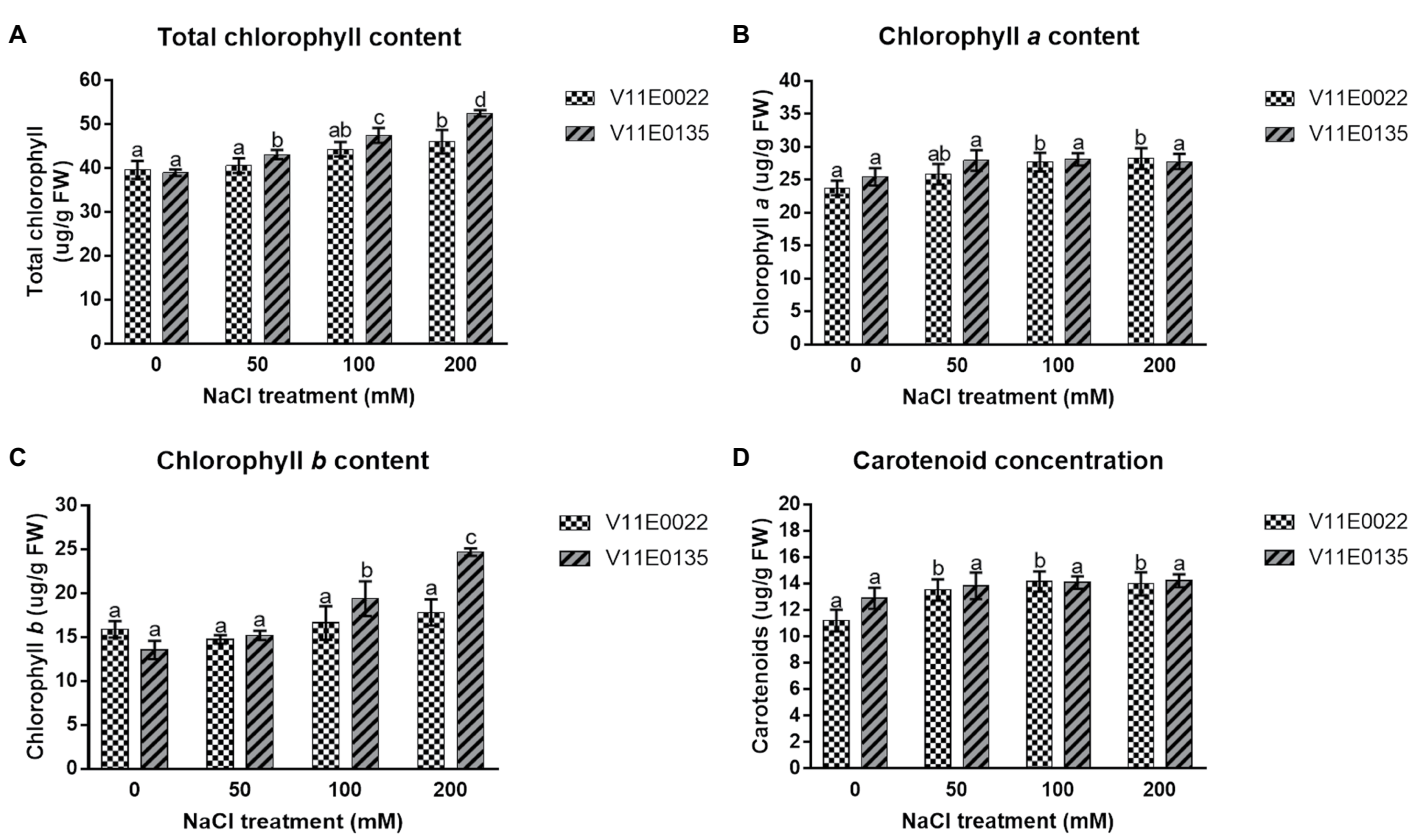
Changes in the photosynthetic pigments under salt stress in leaves of two water dropwort cultivars. **(A)** Total chlorophyll content, **(B)** chlorophyll a content, **(C)** chlorophyll b content and **(D)** carotenoid concentration in the leaves of water dropwort. Means followed by different letters indicate a significant difference (*p* < 0.05) among the four treatments according to the Tukey test. Error bars show mean ± SE.

A zigzag trend of chlorophyll content was found in the leaves under different salt concentrations of all 48 cultivars (Supplementary Table S1). Interestingly, the chlorophyll content was increased in many cultivars of water dropwort. Similarly, the concentration of photosynthetic pigments, including chlorophyll *a* (chl *a*) and chlorophyll *b* (chl *b*) as well as total chlorophyll (chl *a*+*b*) and carotenoids (Car), was higher in the salt-treated plants compared to the non-treated plants of both selected sensitive and tolerant cultivars (*p* < 0.05; Figures 2A–D). Specifically compared to the control, the concentration of chl (*a*+*b*) and chl *b* was higher in both cultivars, and maximum concentration was present at 200 mm NaCl treatment. Although higher than in the control situation, a comparable concentration of chl *a* and Car was present in all treatments.

### Lipid Peroxidation and H_2_O_2_ Content

**Figure 3:**
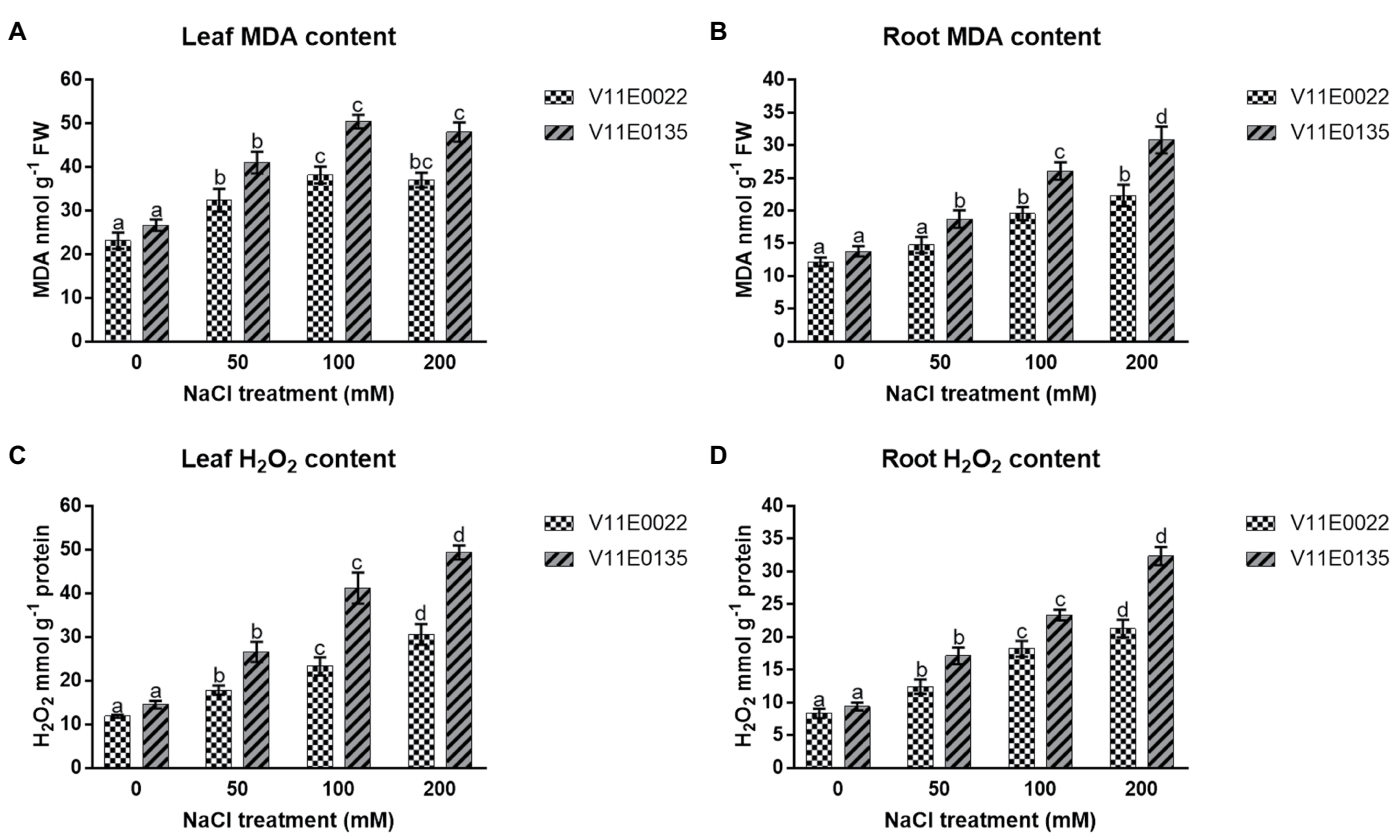
Changes in the lipid peroxidation and ROS in fresh leaves and roots of two water dropwort cultivars under salt stress. **(A)** MDA content in the leaves, **(B)** MDA content in the roots, **(C)** H2O2 content in the leaves and **(D)** H2O2 content in the roots of water dropwort. Means followed by different letters indicate a significant difference (*p* < 0.05) among the four treatments according to the Tukey test. Error bars show mean ± SE.

The salt stress significantly induced lipid peroxidation in terms of MDA content in both leaves and roots of water dropwort cultivars (*p* < 0.05). Moreover, high MDA content was present in V11E0135 compared to the V11E0022. Compared to the control, the MDA content was increased maximally up to 100 mm in leaves of V11E0022 and V11E0135 (Figure 3A), whereas in the roots of both cultivars were found significantly higher under all salt treatments compared to the control (*p* < 0.05; Figure 3B).

The H_2_O_2_ production rate was significantly increased in leaves and roots of both cultivars as compared to the control (*p* < 0.05). Moreover, a significantly higher content of H_2_O_2_ was present in V11E0135 compared to the V11E0022 (*p* < 0.05). Compared to untreated plants, maximum H_2_O_2_ content was present at 200 mm NaCl concentration in leaves and roots of both cultivars (Figures 3C,D).

### Osmolytes and Antioxidant Molecules

**Figure 4:**
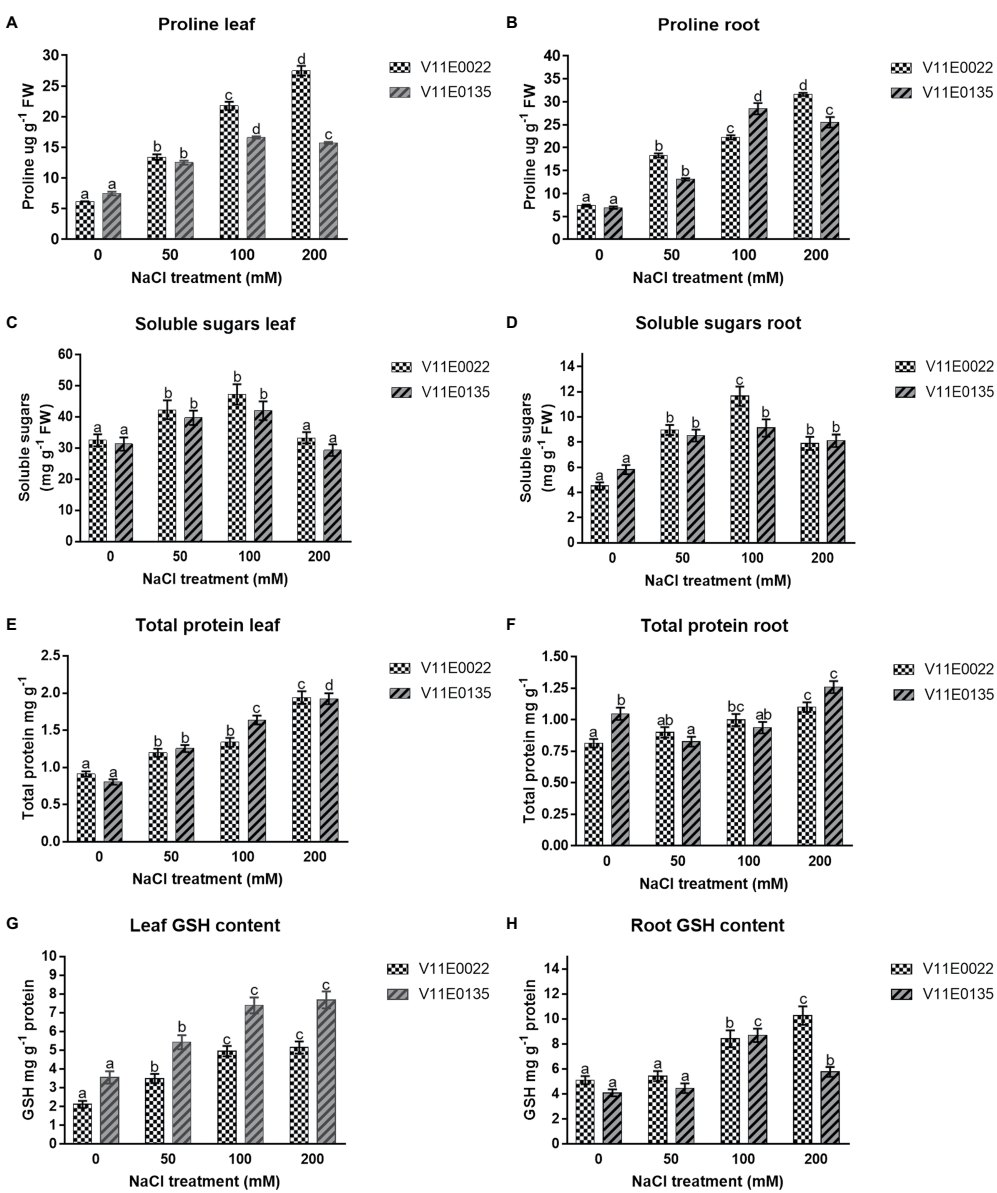
Changes in the content of osmolytes and non-enzymatic antioxidant compounds in fresh leaves and roots of two water dropwort cultivars under salt stress. **(A)** Proline content in the leaves, **(B)** proline content in the roots, **(C)** soluble sugars content in the leaves, **(D)** soluble sugars content in the roots, **(E)** total protein content in the leaves, **(F)** total protein content in the roots, **(G)** reduced glutathione (GSH) content in the leaves and **(H)** GSH content in the roots of water dropwort. Means followed by different letters indicate a significant difference (*p* < 0.05) among the four treatments according to the Tukey test. Error bars show mean ± SE.

The proline concentration was found higher in V11E0022 compared to V11E0135. The proline content increases significantly in the leaves and roots of V11E0022 in all NaCl treatments compared to the control (*p* < 0.05). The V11E0135 showed a gradual rise in content of proline in leaves and roots up to 100 mm NaCl. Thereafter, a significant decline was observed at 200 mm NaCl (*p* < 0.05; Figures 4A,B). The concentration of soluble sugars was found higher in V11E0022 compared to the V11E0135. In leaves and roots of both cultivars, the concentration of soluble sugar was found to be significantly increasing up to 100 mm NaCl concentration compared to the control (*p* < 0.05; Figures 4C,D).

The results showed that the protein content was increased with the increasing of NaCl concentration in both selected cultivars. A significant difference was observed in protein concentration with the increasing salt concentration in leaves and roots of V11E0022 as compared to the control (*p* < 0.05; Figures 4E,F). In contrast, the leaves of V11E0135 showed a significant increase in all treatments (*p* < 0.05; Figure 4E). However, protein content in roots of V11E0135 was significantly decreased by 21.09% at 50 mm in comparison with its respective control (*p* < 0.05), and thereafter increased at 100 and 200 mm NaCl concentrations (Figure 4F).

GSH content was increased in both leaves and roots of V11E0022 with the increasing NaCl concentration, and the highest GSH content was found at 200 mm NaCl concentration (Figures 4G,H). The V11E0022 showed higher GSH content than the V11E0135 in roots. Interestingly, the leaves of V11E0135 showed higher content of GSH compared to its counterpart, but its roots showed maximum GSH content at 100 mm NaCl concentration.

### Antioxidant Enzymes

**Figure 5:**
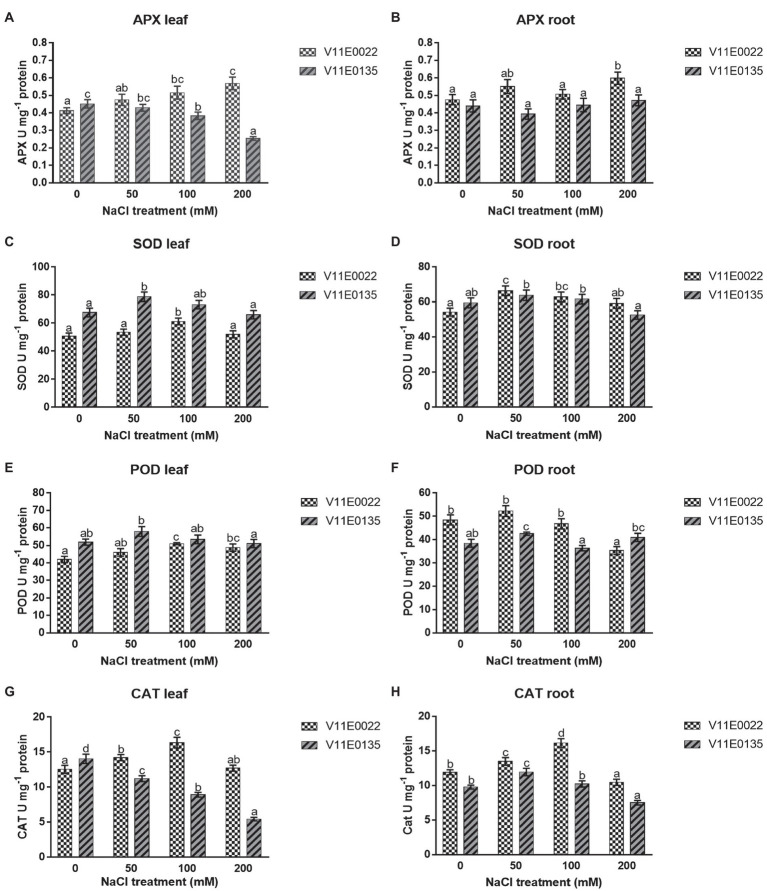
Changes in activities of antioxidant enzymes in fresh leaves and roots of two water dropwort cultivars under salt stress. **(A)** APX activity in the leaves, **(B)** APX activity in the roots, **(C)** SOD activity in the leaves, **(D)** SOD activity in the roots, **(E)** POD activity in the leaves, **(F)** POD activity in the roots, **(G)** CAT activity in the leaves and **(H)** CAT activity in the roots of water dropwort. Means followed by different letters indicate a significant difference (*p* < 0.05) among the four treatments according to the Tukey test. Error bars show mean ± SE.

APX activity was found higher in V11E0022 compared to the V11E0135. The activity increased significantly with the increasing salt concentration in leaves and roots of V11E0022 compared to its counterpart (*p* < 0.05; Figures 5A,B). In contrast, the APX activity decreased gradually in leaves of V11E0135 with the increasing salt concentration (Figure 5A). However, APX activity decreased up to 10.68% at 50 mm NaCl concentration in roots; nevertheless, comparatively higher activity was observed at 100 and 200 mm NaCl concentration (*p* > 0.05; Figure 5B).

A similar pattern was observed for SOD and POD in both cultivars under different NaCl treatments. Compared to the control, the activities of SOD and POD were increased significantly up to 100 mm in the leaves of V11E0022 (*p* < 0.05; Figures 5C,E), while decreased under 50 mm NaCl in the leaves of V11E0135. Furthermore, SOD and POD activities were decreased after 50 mm NaCl in roots of both cultivars. Interestingly, the POD activity in V11E0135 at 200 mm was 6.53% higher than the control (Figures 5D,F). When compared based on the difference in activities with their respective controls, V11E0022 was found comparatively higher than V11E0135 in both antioxidant enzyme.

A significantly higher CAT activity was observed in V11E0022 compared to the V11E0135 (*p* < 0.05). The CAT activity increased up to 100 mm NaCl concentration in leaves and roots of V11E0022 (Figures 5G,H). In contrast, the CAT activity was decreased significantly with the increasing salt concentration in leaves of V11E0135. In contrast, the CAT activity was found significantly higher in roots at 50 mm and thereafter decreased gradually at 100 and 200 mm NaCl concentrations (*p* < 0.05; Figures 5G,H).

## Discussion

Salinity is a major abiotic stress that significantly affects the plant growth by causing osmotic stress, and inducing ionic and nutrient imbalance. Such imbalances adversely affect different physiological and biochemical mechanisms related to the plant growth and development (Zhang et al., 2013). The present study investigated the phenotypic effects of salt stress on 48 water dropwort cultivars at different NaCl concentration (0–200 mm). The study proposed the tolerant and sensitive cultivars based on their performance against salt stress, and different components of the antioxidant defense system depicted the salt tolerance mechanism in selected sensitive and tolerant cultivars of water dropwort.

The results of the present study show that the plant growth (total height, stem and root lengths, and number of branches and leaves) was decreased significantly with the increasing NaCl concentration in all 48 cultivars of water dropwort, indicating that salt stress suppressed their growth. The growth reduction in V11E0135 was very pronounced in comparison with other cultivars, whereas V11E0022 showed better adaptation as compared to others. Similar studies were previously conducted on different plants also support our findings (Shaheen et al., 2013; Okkaoğlu et al., 2015; Menezes et al., 2017; Rahneshan et al., 2018; Sahin et al., 2018). Furthermore, the fresh and dry biomass of shoot and root was significantly decreased in both selected cultivars of water dropwort under all treatments of NaCl, whereas V11E0135 showed more reduction than the V11E0022. Previous studies on different plants showed the reduction of fresh and dry weights of root and shoot under NaCl stress (Inal et al., 2009; Shaheen et al., 2013; Kapoor and Pande, 2015). According to Meriem et al. (2014), a higher reduction in biomass was observed in sensitive cultivars than tolerant cultivar of coriander under different NaCl treatments. It is suggested that this decrease in the length and biomass of water dropwort could be due to the negative effect of NaCl treatment. The salinity increases the osmotic stress that inhibits absorption and transport of water. This inhibition leads to hormones-induced sequential reactions, which can reduce the stomatal opening, CO_2_ assimilation, and photosynthetic rate (Odjegba and Chukwunwike, 2012; Menezes et al., 2017; Sarker and Oba, 2020b). Another reason for reduction in growth might be the diversion of energy from growth to the homeostasis of salinity stress and a reduction in carbon gains (Atkin and Macherel, 2009; Sarker and Oba, 2020b).

Based on all phenotypic results of the current study, it is suggested that the decrease in growth and biomass could be due to the adverse effects of salinity on cell division and elongation. Moreover, salinity also causes the nutrient imbalance, overproduction of ROS, and inhibition of enzymatic activities, which significantly affect the cellular components and biological membranes and cause a decrease in biomass production (Ali et al., 2017; Alzahrani et al., 2019).

A high concentration of NaCl affects photosynthesis, and the exposure to salt stress for longer time causes a reduction in biosynthesis of chlorophyll protein-lipid complex (Akbari Ghogdi et al., 2012). Different opinions about the salinity effect on the chlorophyll content have been reported, and among these many studies have reported a significantly decreased in chlorophyll content under salt stress (Çelik and Atak, 2012; Meriem et al., 2014; Sharif et al., 2017). However, the results of higher chlorophyll *a*, *b*, and the total chlorophyll content in the present study are in agreement with the studies previously conducted on amaranth (*Amaranthus tricolor*), sugar beet, and cabbage (Wang and Nii, 2000; Jamil et al., 2007). These mentioned studies suggest that an increase in the chlorophyll content under salt stress could be due to the increased number of chloroplasts. Similar results on lettuce suggest that the increased chlorophyll content could be due to the accumulation of NaCl in the chloroplast (Ekinci et al., 2012). According to Jamil et al. (2007), tolerance of photosystem II (PSII) to high salt stress and increased chlorophyll content had played important in salinity tolerance of cabbage and sugar beet; therefore, it might be possible that PSII can play important role in salinity stress tolerance of water dropwort, but it needs further studies. Our results indicate that increased in chlorophyll content under salt stress could be helpful to grow water dropwort in the saline soils. Carotenoid is a type of antioxidant which helps in developing tolerance against salt stress in plants by reducing the free oxygen radicals (Ali et al., 2017). In the current study, the carotenoid concentration was slightly increased under NaCl stress in both selected cultivars compared to the control, where V11E0022 showed relatively high concentration. A previous study also reported an increase in the carotenoids concentration under salt stress (Çelik and Atak, 2012). As an antioxidant, carotenoids help reducing the singlet oxygen for preventing the oxidative damage.

Salt stress induces the concentration of Na^+^ ions in the plant cells. The excess accumulation of Na^+^ in the cytosol is concomitant with salt-induced K^+^ efflux, and the cytosolic K^+^/Na^+^ ratio decreases dramatically under salinity stress conditions (Silva et al., 2015; Sarker and Oba, 2020b). This might be linked with the fact that Na^+^ enters the roots passively through voltage-independent or weakly voltage-dependent nonselective cation channels. This could be also linked with other Na^+^ transporters, such as members of the high-affinity K^+^ transporters. Thus, increase in the level of external Na^+^ will sensibly rise the accumulation of Na^+^ in the plants with concomitant decrease in K^+^ uptake (Odjegba and Chukwunwike, 2012; Silva et al., 2015; Sarker and Oba, 2020b). Several authors have suggested that low uptake of Na^+^ and high uptake of K^+^ signifies salinity tolerance in higher plants (Yassin et al., 2019). In the present study, a significant increase in Na^+^ uptake and decrease in the K^+^ uptake was observed in leaves and roots of both cultivars with increasing NaCl concentration, whereas the leaves showed more ionic uptake than the roots. Furthermore, high uptake of Na^+^ was observed in the V11E0135 cultivar as compared to V11E0022. Previous studies on pistachio, paper mulberry, and wheat also showed a higher uptake of Na^+^ in sensitive cultivars, which support the findings of the current study (Zhang et al., 2013; Rahneshan et al., 2018; Yassin et al., 2019). Roots and shoots of the carrot and amaranth also showed high Na^+^ uptake and decreased K^+^ uptake under salt stress; moreover, the roots showed a considerably low concentration of Na^+^ and K^+^ compared to the shoots (Inal et al., 2009; Menezes et al., 2017). In the current study, we found the higher level of Na^+^ in the leaves as compared to the roots, and the reason is that leaves are most porn to Na^+^ than roots because Na^+^ and Cl^−^ accumulate more in shoots than the roots. Roots maintain constant level of NaCl over time and can regulate NaCl levels by export to the shoots or to the soil. Na^+^ is transported to shoots in the rapidly moving transpiration stream in the xylem (Tester and Davenport, 2003). Different studies reported that salinity-tolerant plants either limit the excess salt in the vacuole or compartmentalize essential ions in different plant tissues. This compartmentalization of Na^+^ into the vacuoles or its efflux across the plasma membrane is controlled by the expression and activity of Na^+^/H^+^ antiporters, V-type H^+^-ATPase, and H^+^-PPase, which ultimately increased the K^+^/Na^+^ ratio (Türkan and Demiral, 2009; Tsujii et al., 2019). The results of these parameters in the present study suggest that a low-level uptake of Na^+^ in V11E0022 might be due to these antiporters and membrane transporters, which can help to stand against the salt stress. RWC is considered a useful and reliable parameter to check the salt stress (Sharif et al., 2017; Sarker and Oba, 2020b). V11E0022 showed the low uptake of Na^+^ and less reduction in K^+^ under salt stress enabled the plant to retain more RWC compared to the V11E0135. Thus, V11E0022 is able to keep a high salt concentration and can absorb more water and consequently has high RWC to adjust osmotic pressure.

Lipid peroxidation is an indicator of oxidative damage caused by salt stress, and the higher concentration of MDA under stress represents the degree of cell membrane damage and it is a common physiological indicator for evaluating plant exposed to biotic or abiotic stress (Sarker and Oba, 2020b). In general, the salt-tolerant cultivars exhibit less lipid peroxidation and ROS production (H_2_O_2_) compared to their sensitive counterparts, which is attributed to efficient protection mechanisms and predominantly high scavenging capacity of the tolerant cultivar (Yassin et al., 2019). The results of the present study showed a significantly higher concentration of MDA and H_2_O_2_ in V11E0135 compared to V11E0022 under salt stress, which is in agreement with the previous studies on different plants (Wang et al., 2009; Shafeiee and Ehsanzadeh, 2019; Yassin et al., 2019; Sarker and Oba, 2020b). In the current study, a minor decrease in MDA concentration was observed at 200 mm NaCl in the leaves of both cultivars. 200 mm NaCl might induces salt stress-related genes in the leaves of water dropwort (Kumar et al., 2020). Sustained decreases in MDA accumulation might be due to the activation of PSII core proteins and Rubisco (Morales and Munné-Bosch, 2019). A negative correlation between MDA content and electron transport was described by Morales and Munné-Bosch, which implies a feedback of PSII and lower MDA in salt-stressed plants. From the current study, we assume that the higher accumulation of MDA and H_2_O_2_ has severely affected the phenotype of V11E0135 as compared to the V11E0022. To cope with this situation, the plants have a defense system in the form of osmolytes, antioxidant molecules, and antioxidant enzymes.

To regulate the osmotic potential, different compatible solute, such as proline, soluble sugars, proteins, and GSH, was accumulated in plants. The higher level of these compounds helps in selecting the tolerant cultivar under stress conditions (Torabi et al., 2013; Sharif et al., 2017; Sarker and Oba, 2019). Accumulation of proline and soluble sugars under stress conditions protects the cell by maintaining the osmotic strength of cytosol with that of vacuole and external environment. In addition to its osmoprotection role, proline is prominently used against ROS as well as provide protection to enzymes and stabilize their structures (Rahneshan et al., 2018; Alzahrani et al., 2019). Previous studies reported that the salinity tolerant cultivars of canola, coriander, and tobacco showed an increment in the proline content and soluble sugars, which is in agreement with the current results (Çelik and Atak, 2012; Meriem et al., 2014; Sharif et al., 2017). Proline content increased with increasing NaCl stress in both roots and leaves of tolerant cultivar, whereas, in sensitive cultivar it starts to decrease after 100 mm NaCl. The decrease at 200 mm NaCl stress might be due to the low activity of enzymes (P5CS and glutamine dehydrogenase) of the proline biosynthetic pathway in V11E0135 (Chun et al., 2018). Another reason might be proline dehydrogenase (ProDH), which is one of the key enzymes that regulates proline accumulation. Therefore, it might be possible that ProDH genes (*ProDH1* and *ProDH2*) expression has been decreased at higher concentration of NaCl in V11E0135 (Funck et al., 2010; Chun et al., 2018).

The reason for the increment of soluble sugars might be the higher enzymatic activities that help in the regulation of cellular structures and functions through the interaction with macromolecules (Sharif et al., 2017; Ibrahimova et al., 2019). The tolerant cultivars retain more water due to proline and sugars, and the present study also showed that the higher RWC of V11E0022 is due to the elevated concentrations of proline and soluble sugars, which improves the osmotic adjustment in water dropwort. Different studies revealed that the salt stress reduced the RWC in the plants, and a direct consequence of higher osmolytes in tolerant cultivar is the maintenance of comparatively higher RWC (Karlidag et al., 2009; Alzahrani et al., 2019; Shafeiee and Ehsanzadeh, 2019).

Furthermore, proteins act as osmotin and their accumulation play a potential role developing tolerance against the salt stress (Qados, 2011; Zhang et al., 2013; Sarker et al., 2018). Results of the current study showed that the protein content in leaves and roots of both cultivars was increased significantly under salt stress. The current results are in agreement with the previous studies conducted on *Vicia faba*, *Broussonetia papyrifera*, and *Amaranthus tricolor*, which showed an increment in the protein content in both roots and shoots under salt stress (Qados, 2011; Zhang et al., 2013; Sarker et al., 2018). According to Yan et al. (2018), the synthesis and accumulation of GSH can improve tolerance against biotic and abiotic stresses. Moreover, GSH helps in ROS scavenging by detoxifying the superoxide and hydroxyl radical (Ashraf, 2009; Hussain et al., 2016). In the present study, a higher level of GSH was found in both cultivars of water dropwort under salt stress compared to the control. The roots of V11E0022 showed a higher level of GSH in comparison with its counterparts, it could help in developing salt tolerance. Likewise, studies carried out on wheat and onion showed the positive effect of GSH by improving cell viability under salt stress (Aly-Salama and Al-Mutawa, 2009; Ahanger et al., 2019). Surprisingly, the leaves of V11E0135 showed higher GSH than the V11E0022, and this increase in leaves could be due to respiration, which plays a vital role in biosynthesis of GSH. Metabolites, such as glycine, are produced during the respiration that could be used in the biosynthesis of GSH (Aly-Salama and Al-Mutawa, 2009). This higher GSH content is concomitant with a higher respiration rate in V11E0135. All these osmolytes and antioxidants might be responsible for osmotic adjustment as well as the reduction of ROS and oxidative stress, which enhance the tolerance of V11E0022 under salt stress.

Higher activities of antioxidant enzymes (SOD, POD, CAT, and APX) provide tolerance against salt stress by scavenging ROS, and the tolerant plants possess higher enzyme activities than the sensitive counterparts (Ali et al., 2017; Polash et al., 2019). In a defense mechanism, the first line of defense is SOD that transforms the superoxides into H_2_O_2_. Thereafter, CAT further converts this H_2_O_2_ into H_2_O and oxygen. Likewise, APX converts the H_2_O_2_ into H_2_O. In addition to these, POD is also used to scavenge H_2_O_2_ from the chloroplast efficiently (Jalali-e-Emam et al., 2011; Polash et al., 2019). Similarly, GR converts the glutathione (GSSG) into reduced GSH which regulates the ROS removal (Elsawy et al., 2018; Polash et al., 2019). Previous studies reported the higher activities of APX, SOD, POD, and CAT in response to salinity in tomato, cabbage, amaranth, and wheat (Li, 2009; Ali et al., 2017; Sahin et al., 2018; Sarker and Oba, 2020b).

Considering biomass and growth as the indicators for salt tolerance, we deduce that V11E0022 is more tolerant than the V11E0135, and this high tolerance could be attributed to better antioxidant enzyme activities *viz* SOD, POD, CAT, and APX, which reduced the H_2_O_2_ and lipid peroxidation level in roots and leaves. SOD and POD of V11E0022 showed higher activity up to 100 mm in the leaves, whereas V11E0135 starts to decrease after 50 mm NaCl. The activity of SOD and POD in roots of both cultivars decreased after 50 mm NaCl, but comparatively higher activities were found in V11E0022. APX activity in the leaves of V11E0022 was increased with increasing NaCl concentration, whereas V11E0135 showed inverse relation with salt stress. Similarly, APX activity in roots was also found significantly higher in V11E0022 in comparison with V11E0135. CAT showed higher activities up to 100 mm in both leaves and roots of V11E0022; however, leaves of V11E0135 showed decrease in CAT activity with increasing NaCl concentration, whereas roots start to decrease after 50 mm NaCl. The observation of augmented antioxidant capacity of water dropwort up to 100 mm NaCl stress. Previous studies also reported the decreased antioxidant capacities after 100 and 150 mm NaCl stress in *Vigna unguiculata*, *Brassica juncea, Oryza sativa*, *Morus alba*, *Broussonetia papyrifera* and many other plants (Verma and Mishra, 2005; Ahmad et al., 2010; Maia et al., 2010; Zhang et al., 2013; García-Caparrós et al., 2019). Moreover, the present study also suggests that the different parts of water dropwort may behave differently against the salt stress, which depend on the type of cellular metabolism of the plant part. The findings of antioxidant capacity also reveal that APX and CAT could be efficient markers for understanding the potential defense mechanisms of water dropwort under NaCl stress conditions compared to other enzymes.

## Conclusion

Based on the phenotypic and physiological studies, we found that V11E0022 cultivar is tolerance against salt stress among the 48 cultivars, whereas V11E0135 is the most sensitive. Moreover, the tolerance of water dropwort could be due to the higher content of osmolytes and antioxidants, and better activities of APX, SOD, POD, and CAT, which reduced the level of H_2_O_2_, and MDA in roots and leaves of water dropwort. Comparatively higher K^+^/Na^+^ ratio and higher concentration of proline and soluble sugars, which acts as osmoregulators helped in retaining higher water content in V11E0022. Based on the antioxidant defense system, it is suggested that this cultivar could efficiently tolerate the salt stress up to 100 mm NaCl. Furthermore, proline, GSH, APX, and CAT could play efficient roles in water dropwort under NaCl stress conditions compared to others and help to understand the salinity tolerance mechanism in water dropwort.

## Data Availability Statement

The raw data supporting the conclusions of this article will be made available by the authors, without undue reservation.

## Author Contributions

All authors contributed to the manuscript. Conceptualization and funding acquisition: HH and WK. Data curation, investigation, validation, and writing—original draft: SK. Methodology: SK, XH, QJ, and ZL. Writing—review and editing: SK, GL, and HH. All authors have read and agreed to the published version of the manuscript.

### Conflict of Interest

The authors declare that the research was conducted in the absence of any commercial or financial relationships that could be construed as a potential conflict of interest.

## References

[ref1] Ahanger M. A. Qin C. Begum N. Maodong Q. Dong X. X. El-Esawi M.. (2019). Nitrogen availability prevents oxidative effects of salinity on wheat growth and photosynthesis by up-regulating the antioxidants and osmolytes metabolism, and secondary metabolite accumulation. BMC Plant Biol. 19:479. 10.1186/s12870-019-2085-3, PMID: 31703619 PMC6839093

[ref2] Ahmad P. Jaleel C. A. Sharma S. (2010). Antioxidant defense system, lipid peroxidation, proline-metabolizing enzymes, and biochemical activities in two *Morus alba* genotypes subjected to NaCl stress. Russ. J. Plant Physiol. 57, 509–517. 10.1134/S1021443710040084

[ref3] Akbari Ghogdi E. Izadi-Darbandi A. Borzouei A. (2012). Effects of salinity on some physiological traits in wheat (*Triticum aestivum* L.) cultivars. Indian J. Sci. Technol. 5, 1901–1906. 10.17485/ijst/2012/v5i1.23

[ref4] Ali Q. Daud M. K. Zulqurnain M. Ali S. (2017). Seed priming by sodium nitroprusside improves salt tolerance in wheat (*Triticum aestivum* L.) by enhancing physiological and biochemical parameters. Plant Physiol. Biochem. 119, 50–58. 10.1016/j.plaphy.2017.08.010, PMID: 28843888

[ref5] Aly-Salama K. H. Al-Mutawa M. M. (2009). Glutathione-triggered mitigation in salt-induced alterations in plasmalemma of onion epidermal cells. Int. J. Agric. Biol. 11, 639–642.

[ref6] Alzahrani S. M. Alaraidh I. A. Migdadi H. Alghamdi S. Altaf Khan M. Ahmad P. (2019). Physiological, biochemical, and antioxidant properties of two genotypes of *Vicia faba* grown under salinity stress. Pak. J. Bot. 51, 786–798. 10.30848/PJB2019-3(3)

[ref7] Ashraf M. (2009). Biotechnological approach of improving plant salt tolerance using antioxidants as markers. Biotechnol. Adv. 27, 84–93. 10.1016/j.biotechadv.2008.09.003, PMID: 18950697

[ref8] Atkin O. K. Macherel D. (2009). The crucial role of plant mitochondria in orchestrating drought tolerance. Ann. Bot. 103, 581–597. 10.1093/aob/mcn094, PMID: 18552366 PMC2707344

[ref9] Beers R. F. Sizer I. W. (1952). A spectrophotometric method for measuring the breakdown of hydrogen peroxide by catalase. J. Biol. Chem. 195, 133–140. 10.1016/S0021-9258(19)50881-X, PMID: 14938361

[ref10] Çelik Ö. Atak Ç. (2012). The effect of salt stress on antioxidative enzymes and proline content of two Turkish tobacco varieties. Turk. J. Biol. 36, 339–356. 10.3906/biy-1108-11

[ref11] Chan E. W. C. Wong S. K. Chan H. T. (2017). Ulam herbs of *Oenanthe javanica* and *Cosmos caudatus*: an overview on their medicinal properties. J. Nat. Remedies 16, 137–147. 10.18311/jnr/2016/8370

[ref12] Chance B. Maehly A. C. (1955). Assay of catalases and peroxidases. Methods Enzymol. 2, 764–775. 10.1016/S0076-6879(55)02300-8 13193536

[ref13] Chun S. C. Paramasivan M. Chandrasekaran M. (2018). Proline accumulation influenced by osmotic stress in arbuscular mycorrhizal symbiotic plants. Front. Microbiol. 9:2525. 10.3389/fmicb.2018.02525 30459731 PMC6232873

[ref14] Colomer-Winter C. Flores-Mireles A. L. Baker S. P. Frank K. L. Lynch A. J. L. Hultgren S. J.. (2018). Manganese acquisition is essential for virulence of *Enterococcus faecalis*. PLoS Pathog. 14:e1007102. 10.1371/journal.ppat.1007102, PMID: 30235334 PMC6147510

[ref15] Dai W. Wang M. Gong X. Liu J. H. (2018). The transcription factor *FcWRKY40* of *Fortunella crassifolia* functions positively in salt tolerance through modulation of ion homeostasis and proline biosynthesis by directly regulating *SOS2* and *P5CS1* homologs. New Phytol. 219, 972–989. 10.1111/nph.15240, PMID: 29851105

[ref16] Ekinci M. Yildirim E. Dursun A. Turan M. (2012). Mitigation of salt stress in lettuce (*Lactuca sativa* L. var. Crispa) by seed and foliar 24-epibrassinolide treatments. HortScience 47, 631–636. 10.21273/HORTSCI.47.5.631

[ref17] Elsawy H. I. A. Mekawy A. M. M. Elhity M. A. Abdel-dayem S. M. Abdelaziz M. N. Assaha D. V. M.. (2018). Differential responses of two Egyptian barley (*Hordeum vulgare* L.) cultivars to salt stress. Plant Physiol. Biochem. 127, 425–435. 10.1016/j.plaphy.2018.04.012, PMID: 29684827

[ref18] Funck D. Eckard S. Müller G. (2010). Non-redundant functions of two proline dehydrogenase isoforms in *Arabidopsis*. BMC Plant Biol. 10:70. 10.1186/1471-2229-10-70, PMID: 20403182 PMC3095344

[ref19] García-Caparrós P. Hasanuzzaman M. Lao M. T. (2019). “Oxidative stress and antioxidant defense in plants under salinity,” in Reactive Oxygen, Nitrogen and Sulfur Species in Plants. eds. Hasanuzzaman M. Fotopoulos V. Nahar K. Fujita M. (Hoboken: Wiley), 291–309.

[ref20] Gharsallah C. Fakhfakh H. Grubb D. Gorsane F. (2016). Effect of salt stress on ion concentration, proline content, antioxidant enzyme activities and gene expression in tomato cultivars. AoB Plants 8:plw055. 10.1093/aobpla/plw055, PMID: 27543452 PMC5091694

[ref22] Hasanuzzaman M. Oku H. Nahar K. Bhuyan M. H. M. B. Al J. Baluska F.. (2018). Nitric oxide-induced salt stress tolerance in plants: ROS metabolism, signaling, and molecular interactions. Plant Biotechnol. Rep. 12, 77–92. 10.1007/s11816-018-0480-0

[ref23] Hoagland D. R. Arnon D. I. (1950). The Water-Culture Method for Growing Plants Without Soil. 2nd *Edn*. *Vol*. 347. California: Agricultural Experiment Station, 1–32.

[ref24] Hussain S. Khan F. Hussain H. A. Nie L. (2016). Physiological and biochemical mechanisms of seed priming-induced chilling tolerance in rice cultivars. Front. Plant Sci. 7:116. 10.3389/fpls.2016.00116, PMID: 26904078 PMC4746480

[ref25] Ibrahimova U. F. Mammadov A. C. Feyziyev Y. M. (2019). The effect of NaCl on some physiological and biochemical parameters in *Triticum aestivum* L. genotypes. Plant Physiol. Rep. 24, 370–375. 10.1007/s40502-019-00461-z

[ref26] Inal A. Gunes A. Pilbeam D. J. Kadloglu Y. K. Eraslan F. (2009). Concentrations of essential and nonessential elements in shoots and storage roots of carrot grown in NaCl and Na_2_SO_4_ salinity. X-Ray Spectrom. 38, 45–51. 10.1002/xrs.1104

[ref27] Islam F. Wang J. Farooq M. A. Yang C. Jan M. Mwamba T. M.. (2019). “Rice responses and tolerance to salt stress,” in Advances in Rice Research for Abiotic Stress Tolerance. eds. Hasanuzzaman M. Fujita M. Nahar K. Biswas J. (Cambridge: Woodhead Publishing), 791–819.

[ref28] Jalali-e-Emam S. M. S. Alizadeh B. Zaefizadeh M. Zakarya R. A. Khayatnezhad M. (2011). Superoxide dismutase (SOD) activity in NaCl stress in salt-sensitive and salt-tolerance genotypes of colza (*Brassica napus* L.). Middle-East J. Sci. Res. 7, 7–11.

[ref29] Jamil M. Rehman S. Rha E. S. (2007). Salinity effect on plant growth, PSII photochemistry and chlorophyll content in sugar beet (*Beta vulgaris* L.) and cabbage (*Brassica oleracea* capitata L.). Pak. J. Bot. 39, 753–760.

[ref30] Jeon H. R. Abd El-Aty M. Cho S. K. Choi J. Kim K. Park R.. (2007). Multiresidue analysis of four pesticide residues in water dropwort (*Oenanthe javanica*) via pressurized liquid extraction, supercritical fluid extraction, and liquid–liquid extraction and gas chromatographic determination. J. Sep. Sci. 30, 1953–1963. 10.1002/jssc.200600548, PMID: 17638354

[ref31] Jiang Q. Wang F. Tan H. W. Li M. Y. Xu Z. S. Tan G. F.. (2015). De novo transcriptome assembly, gene annotation, marker development, and miRNA potential target genes validation under abiotic stresses in *Oenanthe javanica*. Mol. Gen. Genomics 290, 671–683. 10.1007/s00438-014-0953-y, PMID: 25416420

[ref32] Kapoor N. Pande V. (2015). Effect of salt stress on growth parameters, moisture content, relative water content and photosynthetic pigments of fenugreek variety RMt-1. J. Plant Sci. 10, 210–221. 10.3923/jps.2015.210.221

[ref33] Karlidag H. Yildirim E. Turan M. (2009). Salicylic acid ameliorates the adverse effect of salt stress on strawberry. Sci. Agric. 66, 180–187. 10.1590/S0103-90162009000200006

[ref34] Khan A. Anwar Y. Hasan M. Iqbal A. Ali M. Alharby H.. (2017). Attenuation of drought stress in *Brassica* seedlings with exogenous application of Ca^2+^ and H_2_O_2_. Plan. Theory 6:20. 10.3390/plants6020020, PMID: 28505096 PMC5489792

[ref35] Kumar S. Li G. Huang X. Ji Q. Zhou K. Hou H.. (2021). Phenotypic, nutritional, and antioxidant characterization of blanched *Oenanthe javanica* for preferable cultivar. Front. Plant Sci. 12:639639. 10.3389/fpls.2021.639639 33679854 PMC7933200

[ref36] Kumar S. Li G. Yang J. Huang X. Ji Q. Zhou K.. (2020). Investigation of an antioxidative system for salinity tolerance in *Oenanthe javanica*. Antioxidants 9:940. 10.3390/antiox9100940, PMID: 33019501 PMC7601823

[ref37] Lee J. H. Kim H. R. (2009). Influence of pretreatments on the dehydration characteristics during vacuum drying of water dropwort (*Oenanthe javanica* DC.). J. Food Process. Preserv. 34, 397–413. 10.1111/j.1745-4549.2008.00319.x

[ref38] Li-ping L. Xiao-hua L. Hong-bo S. Zhao-Pu L. Ya T. Quan-suo Z.. (2015). Ameliorants improve saline-alkaline soils on a large scale in northern Jiangsu Province, China. Ecol. Eng. 81, 328–334. 10.1016/j.ecoleng.2015.04.032

[ref39] Li Y. (2009). Physiological responses of tomato seedlings (*Lycopersicon esculentum*) to salt stress. Mod. Appl. Sci. 3, 171–176. 10.5539/mas.v3n3p171

[ref40] Lu C. Li X. (2019). A review of *Oenanthe javanica* (Blume) DC. as traditional medicinal plant and its therapeutic potential. Evidence-Based Complement. Altern. Med. 2019, 1–17. 10.1155/2019/6495819 PMC6463588 31057651

[ref41] Maia J. M. de Macedo C. E. C. Voigt E. L. Freitas J. B. S. Silveira J. A. G. (2010). Antioxidative enzymatic protection in leaves of two contrasting cowpea cultivars under salinity. Biol. Plant. 54, 159–163. 10.1007/s10535-010-0026-y

[ref42] McCord J. M. Fridovich I. (1969). Superoxide dismutase an enzymic function for erythrocuprein (hemocuprein). J. Biol. Chem. 244, 6049–6055. 10.1016/S0021-9258(18)63504-5, PMID: 5389100

[ref43] Menezes R. V. Azevedo Neto A. D. D. Ribeiro M. D. O. Cova A. M. W. (2017). Growth and contents of organic and inorganic solutes in amaranth under salt stress. Pesqui. Agropecuária Trop. 47, 22–30. 10.1590/1983-40632016v4742580

[ref44] Meriem B. F. Kaouther Z. Chérif H. Tijani M. André B. Meriem B. F.. (2014). Effect of priming on growth, biochemical parameters and mineral composition of different cultivars of coriander (*Coriandrum sativum* L.) under salt stress. J. Stress Physiol. Biochem. 10, 84–109.

[ref45] Morales M. Munné-Bosch S. (2019). Malondialdehyde: facts and artifacts. Plant Physiol. 180, 1246–1250. 10.1104/pp.19.00405, PMID: 31253746 PMC6752910

[ref46] Nakano Y. Asada K. (1981). Hydrogen peroxide is scavenged by ascorbate-specific peroxidase in spinach chloroplasts. Plant Cell Physiol. 22, 867–880. 10.1093/oxfordjournals.pcp.a076232

[ref47] Odjegba V. J. Chukwunwike I. C. (2012). Physiological responses of *Amaranthus hybridus* L. under salinity stress. Indian J. Innov. Dev. 1, 742–748.

[ref48] Okkaoğlu H. Sönmez Ç. Şimşek A. Ö. Bayram E. (2015). Effect of salt stress on some agronomical characteristics and essential oil content of coriander (*Coriandrum sativum* L.) cultivars. J. Appl. Biol. Sci. 9, 21–24.

[ref49] Polash M. A. S. Sakil M. A. Hossain M. A. (2019). Plants responses and their physiological and biochemical defense mechanisms against salinity: a review. Trop. Plant Res. 6, 250–274. 10.22271/tpr.2019.v6.i2.35

[ref50] Qados A. M. S. A. (2011). Effect of salt stress on plant growth and metabolism of bean plant *Vicia faba* (L.). J. Saudi Soc. Agric. Sci. 10, 7–15. 10.1016/j.jssas.2010.06.002

[ref51] Queirós F. Fontes N. Silva P. Almeida D. Maeshima M. Gerós H.. (2009). Activity of tonoplast proton pumps and Na^+^/H^+^ exchange in potato cell cultures is modulated by salt. J. Exp. Bot. 60, 1363–1374. 10.1093/jxb/erp011, PMID: 19213810

[ref52] Rahneshan Z. Nasibi F. Moghadam A. A. (2018). Effects of salinity stress on some growth, physiological, biochemical parameters and nutrients in two pistachio (*Pistacia vera* L.) rootstocks. J. Plant Interact. 13, 73–82. 10.1080/17429145.2018.1424355

[ref53] Sahin U. Ekinci M. Ors S. Turan M. Yildiz S. Yildirim E. (2018). Effects of individual and combined effects of salinity and drought on physiological, nutritional and biochemical properties of cabbage (*Brassica oleracea var. capitata*). Sci. Hortic. 240, 196–204. 10.1016/j.scienta.2018.06.016

[ref54] Sanower-Hossain M. (2019). Present scenario of global salt affected soils, its management and importance of salinity research. Int. Res. J. Biol. Sci. 1, 1–3.

[ref55] Sarker U. Islam M. T. Oba S. (2018). Salinity stress accelerates nutrients, dietary fiber, minerals, phytochemicals and antioxidant activity in *Amaranthus tricolor* leaves. PLoS One 13:e0206388. 10.1371/journal.pone.0206388, PMID: 30383779 PMC6211690

[ref56] Sarker U. Oba S. (2018). Drought stress effects on growth, ROS markers, compatible solutes, phenolics, flavonoids, and antioxidant activity in *Amaranthus tricolor*. Appl. Biochem. Biotechnol. 186, 999–1016. 10.1007/s12010-018-2784-5, PMID: 29804177

[ref57] Sarker U. Oba S. (2019). Salinity stress enhances color parameters, bioactive leaf pigments, vitamins, polyphenols, flavonoids and antioxidant activity in selected *Amaranthus* leafy vegetables. J. Sci. Food Agric. 99, 2275–2284. 10.1002/jsfa.9423, PMID: 30324618

[ref58] Sarker U. Oba S. (2020a). Nutritional and bioactive constituents and scavenging capacity of radicals in *Amaranthus hypochondriacus*. Sci. Rep. 10:19962. 10.1038/s41598-020-71714-3, PMID: 33203902 PMC7673121

[ref59] Sarker U. Oba S. (2020b). The response of salinity stress-induced *A. tricolor* to growth, anatomy, physiology, non-enzymatic and enzymatic antioxidants. Front. Plant Sci. 11:559876. 10.3389/fpls.2020.559876 33178233 PMC7596248

[ref60] Shafeiee M. Ehsanzadeh P. (2019). Physiological and biochemical mechanisms of salinity tolerance in several fennel genotypes: existence of clearly-expressed genotypic variations. Ind. Crop. Prod. 132, 311–318. 10.1016/j.indcrop.2019.02.042

[ref61] Shaheen S. Naseer S. Ashraf M. Akram N. A. (2013). Salt stress affects water relations, photosynthesis, and oxidative defense mechanisms in *Solanum melongena* L. J. Plant Interact. 8, 85–96. 10.1080/17429145.2012.718376

[ref62] Sharif P. Seyedsalehi M. Paladino O. Damme P. Van Sillanpa M. Sharifi A. A. (2017). Effect of drought and salinity stresses on morphological and physiological characteristics of canola. Int. J. Environ. Sci. Technol. 15, 1859–1866. 10.1007/s13762-017-1508-7

[ref63] Silva E. N. Silveira J. A. G. Rodrigues C. R. F. Viégas R. A. (2015). Physiological adjustment to salt stress in *Jatropha curcas* is associated with accumulation of salt ions, transport and selectivity of K^+^, osmotic adjustment and K^+^/Na^+^ homeostasis. Plant Biol. 17, 1023–1029. 10.1111/plb.12337, PMID: 25865670

[ref64] Singh M. Kumar J. Singh V. P. Prasad S. M. (2014). Plant tolerance mechanism against salt stress: the nutrient management approach. Biochem. Pharmacol. 3:e165. 10.4172/2167-0501.1000e165

[ref65] Soares C. Carvalho M. E. A. Azevedo R. A. Fidalgo F. (2019). Plants facing oxidative challenges—A little help from the antioxidant networks. Environ. Exp. Bot. 161, 4–25. 10.1016/j.envexpbot.2018.12.009

[ref66] Tester M. Davenport R. (2003). Na^+^ tolerance and Na^+^ transport in higher plants. Ann. Bot. 91, 503–527. 10.1093/aob/mcg058, PMID: 12646496 PMC4242248

[ref67] Torabi M. Halim R. A. Mokhtarzadeh A. Miri Y. (2013). “Physiological and biochemical responses of plants in saline environment,” in Crop Biology and Agriculture in Harsh Environments. ed. Roychowdhury R. (Chisinau: Lap Lambert Academic Publishing), 47–80.

[ref68] Tsujii M. Kera K. Hamamoto S. Kuromori T. Shikanai T. Uozumi N. (2019). Evidence for potassium transport activity of *Arabidopsis* KEA1-KEA6. Sci. Rep. 9:10040. 10.1038/s41598-019-46463-7, PMID: 31296940 PMC6624313

[ref69] Türkan I. Demiral T. (2009). Recent developments in understanding salinity tolerance. Environ. Exp. Bot. 67, 2–9. 10.1016/j.envexpbot.2009.05.008

[ref70] Verma S. Mishra S. N. (2005). Putrescine alleviation of growth in salt stressed *Brassica juncea* by inducing antioxidative defense system. J. Plant Physiol. 162, 669–677. 10.1016/j.jplph.2004.08.008, PMID: 16008089

[ref71] Wang W. Kim Y. Lee H. Kim K. Deng X. Kwak S. (2009). Analysis of antioxidant enzyme activity during germination of alfalfa under salt and drought stresses. Plant Physiol. Biochem. 47, 570–577. 10.1016/j.plaphy.2009.02.009, PMID: 19318268

[ref72] Wang Y. Nii N. (2000). Changes in chlorophyll, ribulose bisphosphate carboxylase-oxygenase, glycine betaine content, photosynthesis and transpiration in *Amaranthus tricolor* leaves during salt stress. J. Hortic. Sci. Biotechnol. 75, 623–627. 10.1080/14620316.2000.11511297

[ref73] Yan Z. Ming D. Jin-xia C. U. I. Xian-jun C. Ze-lin W. E. N. Jian-wei Z.. (2018). Exogenous GSH protects tomatoes against salt stress by modulating photosystem II efficiency, absorbed light allocation and H_2_O_2_ scavenging system in chloroplasts. J. Integr. Agric. 17, 2257–2272. 10.1016/S2095-3119(18)62068-4

[ref74] Yang J. Li G. Bishopp A. Heenatigala P. P. M. Hu S. Chen Y.. (2018). A comparison of growth on mercuric chloride for three *Lemnaceae* species reveals differences in growth dynamics that effect their suitability for use in either monitoring or remediating ecosystems contaminated with mercury. Front. Chem. 6:112. 10.3389/fchem.2018.00112 29713627 PMC5911492

[ref75] Yassin M. El Sabagh A. Mekawy A. M. Islam M. Hossain A. Barutcular C.. (2019). Comparative performance of two bread wheat (*Triticum aestivum* L.) genotypes under salinity stress. Appl. Ecol. Environ. Res. 17, 5029–5041. 10.15666/aeer/1702_50295041

[ref76] Zhang J. Zhao X. X. Wang X. Lu W. X. (2015). Effects of cadmium stress on the growth and physiological property of *Oenanthe javanica*. Plant Physiol. J. 51, 1969–1974. 10.13592/j.cnki.ppj.2015.0457

[ref77] Zhang M. Fang Y. Ji Y. Jiang Z. Wang L. (2013). Effects of salt stress on ion content, antioxidant enzymes and protein profile in different tissues of *Broussonetia papyrifera*. S. Afr. J. Bot. 85, 1–9. 10.1016/j.sajb.2012.11.005

