# Supplementary material for: Effect of Salt Stress on Growth, Physiological Parameters, and Ionic Concentration of Water Dropwort (Oenanthe javanica) Cultivars
Source: Front Plant Sci. 2021 Jun 21;12:660409. doi: 10.3389/fpls.2021.660409 (PMC8256277; doi:10.3389/fpls.2021.660409)
Supplement: Supplementary file 1 [file Table_1.DOCX]

**Table S1.** Effect of salt stress on the growth and chlorophyll of 48 water dropwort cultivars. Different letters indicate a significant difference (*P* < 0.05) among the four treatments according to the Tukey test. Values are means ± SE**.**

| **Plant Name** | **NaCl**  **(mM)** | **Plant height (cm)** | **Root length (cm)** | **Stem length (cm)** | **Number of branches** | **Number of leaves** | **Chlorophyll** |
| --- | --- | --- | --- | --- | --- | --- | --- |
| **Liyang**  **(V11E0100)** | 0 | 70.3 ± 3.2^c^ | 29.3 ± 1.2^c^ | 41.0 ± 2.0^c^ | 7 ± 1^b^ | 38 ± 2^b^ | 32.5 ± 1.0^a^ |
|  | 50 | 63.7 ± 2.1^bc^ | 24.3 ± 1.5^b^ | 39.3 ± 1.5^bc^ | 6 ± 0^ab^ | 35 ± 3^b^ | 33.4 ± 1.8^a^ |
|  | 100 | 60.0 ± 3.0^b^ | 22.3 ± 0.6^b^ | 37.7 ± 2.1^b^ | 6 ± 1^ab^ | 27 ± 2^a^ | 34.7 ± 1.9^a^ |
|  | 200 | 53.7 ± 2.1^a^ | 19.7 ± 2.1^a^ | 34.0 ± 1.0^a^ | 5 ± 0^a^ | 25 ± 1^a^ | 33.7 ± 2.2^a^ |
| **Jiangsu**  **(V11E0051)** | 0 | 54.2 ± 2.0^b^ | 15.8 ± 0.8^a^ | 38.3 ± 1.5^b^ | 5 ± 1^b^ | 27 ± 2^b^ | 32.8 ± 0.6^ab^ |
|  | 50 | 51.7 ± 3.2^ab^ | 17.7 ± 1.5^a^ | 34.0 ± 2.0^a^ | 4 ± 1^b^ | 25 ± 2^b^ | 35.5 ± 1.6^b^ |
|  | 100 | 50.3 ± 2.5^ab^ | 16.3 ± 0.6^a^ | 34.0 ± 1.0^a^ | 4 ± 1^b^ | 23 ± 2^b^ | 31.8 ± 1.1^a^ |
|  | 200 | 48.0 ± 1.0^a^ | 15.3 ± 0.6^a^ | 32.7 ± 1.2^a^ | 3 ± 1^a^ | 15 ± 1^a^ | 33.0 ± 1.1^ab^ |
| **Lujiang 98**  **(V11E0036)** | 0 | 63.7 ± 3.1^b^ | 25.0 ± 1.0^b^ | 38.7 ± 1.2^b^ | 6 ± 0^c^ | 37 ± 1^d^ | 27.1 ± 1.1^a^ |
|  | 50 | 56.3 ± 3.2^ab^ | 23.0 ± 1.0^b^ | 34.0 ± 2.0^a^ | 5 ± 0^bc^ | 32 ± 2^c^ | 31.0 ± 1.4^bc^ |
|  | 100 | 52.7 ± 4.0^a^ | 18.3 ± 1.5^a^ | 34.3 ± 2.3^ab^ | 4 ± 0^ab^ | 22 ± 2^b^ | 33.8 ± 1.6^c^ |
|  | 200 | 49.3 ± 1.3^a^ | 17.8 ± 1.6^a^ | 31.5 ± 0.9^a^ | 3 ± 0^a^ | 17 ± 1^a^ | 28.0 ± 1.3^ab^ |
| **Shangrao**  **(V11E0124)** | 0 | 82.0 ± 4.0^c^ | 22.0 ± 1.0^b^ | 60.0 ± 2.0^d^ | 10 ± 1^c^ | 59 ± 3^d^ | 33.1 ± 1.7^a^ |
|  | 50 | 70.3 ± 2.5^b^ | 22.5 ± 1.5^b^ | 48.0 ± 2.0^c^ | 8 ± 2^bc^ | 40 ± 3^c^ | 35.1 ± 1.9^a^ |
|  | 100 | 63.0 ± 4.0^b^ | 21.0 ± 1.0^b^ | 42.0 ± 3.0^b^ | 5 ± 1^ab^ | 30 ± 2^b^ | 36.3 ± 1.5^a^ |
|  | 200 | 50.0 ± 2.6^a^ | 16.0 ± 1.3^a^ | 34.0 ± 1.0^a^ | 3 ± 0^a^ | 15 ± 1^a^ | 36.3 ± 0.9^a^ |
| **Leping (V11E0019)** | 0 | 64.0 ± 2.0^c^ | 24.7 ± 1.5^d^ | 39.3 ± 0.6^b^ | 8 ± 0^b^ | 44 ± 2^b^ | 30.9 ± 1.1^a^ |
|  | 50 | 58.7 ± 4.2^b^ | 19.7 ± 1.2^c^ | 38.0 ± 2.0^b^ | 7 ± 1^b^ | 41 ± 2^b^ | 38.6 ± 1.0^b^ |
|  | 100 | 54.3 ± 4.0^ab^ | 17.0 ± 1.0^b^ | 37.3 ± 2.5^b^ | 5 ± 1^a^ | 25 ± 2^a^ | 37.3 ± 2.0^b^ |
|  | 200 | 49.0 ± 1.0^a^ | 14.3 ± 0.6^a^ | 34.7 ± 1.2^a^ | 4 ± 1^a^ | 24 ± 2^a^ | 31.7 ± 1.9^a^ |
| **Wuhu**  **(V11E0131)** | 0 | 52.7 ± 3.5^b^ | 19.0 ± 1.3^a^ | 33.7 ± 1.2^c^ | 7 ± 1^c^ | 37 ± 1^d^ | 28.4 ± 1.2^a^ |
|  | 50 | 46.7 ± 1.2 ^ab^ | 16.0 ± 1.0^a^ | 30.7 ± 0.6^b^ | 5 ± 1^bc^ | 28 ± 2^c^ | 34.7 ± 1.3^b^ |
|  | 100 | 46.3 ± 2.3^a^ | 18.3 ± 1.0a | 28.0 ± 1.0^ab^ | 4 ± 1^a^ | 23 ± 2^b^ | 37.8 ± 1.6^b^ |
|  | 200 | 45.0 ± 2.0^a^ | 17.7 ± 1.5^a^ | 27.3 ± 1.5^a^ | 4 ± 1^a^ | 15 ± 1^a^ | 37.0 ± 1.5^b^ |
| **Luanjiang**  **shijiao-1**  **(V11E0093)** | 0 | 53.3 ± 3.8^b^ | 19.7 ± 0.6_c_ | 33.7 ± 1.5^b^ | 6 ± 1^b^ | 32 ± 2^b^ | 30.7 ± 1.5^a^ |
|  | 50 | 49.7 ± 3.5^ab^ | 18.0 ± 1.0^bc^ | 31.7 ± 2.1^ab^ | 5 ± 1^b^ | 32 ± 2^b^ | 32.9 ± 1.2^a^ |
|  | 100 | 47.7 ± 1.5^ab^ | 16.3 ± 0.6^ab^ | 31.3 ± 1.5^ab^ | 5 ± 0^b^ | 28 ± 1^b^ | 38.6 ± 2.1^b^ |
|  | 200 | 42.3 ± 2.5^a^ | 14.3 ± 1.0^a^ | 28.0 ± 1.5^a^ | 3 ± 1^a^ | 16 ± 1^a^ | 40.4 ± 1.8^b^ |
| **Hongzhe**  **(V11E0092)** | 0 | 65.3 ± 3.1^c^ | 23.7 ± 1.5^b^ | 41.7 ± 0.6^c^ | 8 ± 1^b^ | 43 ± 3^d^ | 38.8 ± 1.9^ab^ |
|  | 50 | 60.7 ± 3.1^bc^ | 23.3 ± 0.6^b^ | 34.0 ± 2.0^b^ | 7 ± 1^b^ | 35 ± 2^c^ | 42.4 ± 1.6^b^ |
|  | 100 | 54.0 ± 2.0^b^ | 21.7 ± 0.6^b^ | 32.3 ± 1.5^ab^ | 4 ± 1^a^ | 20 ± 2^b^ | 37.2 ± 1.4^a^ |
|  | 200 | 45.0 ± 2.6^a^ | 16.0 ± 1.0^a^ | 29.0 ± 1.7^a^ | 3 ± 1^a^ | 14 ± 1^a^ | 35.0 ± 1.9^a^ |
| **Shaoyan**  **(V11E0022)** | 0 | 68.3 ± 1.5^c^ | 23.0 ± 1.0^c^ | 45.3 ± 0.6^c^ | 8 ± 0^c^ | 63 ± 3^c^ | 34.7 ± 1.5^a^ |
|  | 50 | 61.3 ± 1.5^b^ | 21.0 ± 1.0^bc^ | 40.3 ± 1.2^b^ | 6 ± 1^b^ | 51 ± 2^b^ | 36.8 ± 1.5^a^ |
|  | 100 | 57.0 ± 2.0^ab^ | 19.3 ± 0.6^b^ | 37.7 ± 1.5^ab^ | 5 ± 1^ab^ | 43 ± 2^a^ | 37.5 ± 1.8^b^ |
|  | 200 | 52.8 ± 1.3^a^ | 16.5 ± 0.5^a^ | 36.3 ± 1.5^a^ | 5 ± 1^a^ | 41 ± 2^a^ | 40.6 ± 2.0^a^ |
| **Hefeizhong ye (V11E0135)** | 0 | 58.3 ± 1.5^c^ | 21.3 ± 0.6^c^ | 37.0 ± 1.0^b^ | 7 ± 1^c^ | 64 ± 2^d^ | 33.4 ± 1.7^a^ |
|  | 50 | 46.3 ± 1.2^b^ | 19.0 ± 0.5^b^ | 27.3 ± 0.8^a^ | 4 ± 1^b^ | 27 ± 1^c^ | 36.8 ± 1.4^b^ |
|  | 100 | 41.7 ± 1.5^a^ | 15.0 ± 0.5^a^ | 26.5 ± 0.3^a^ | 2 ± 1^ab^ | 16 ± 1^b^ | 38.9 ± 1.4^b^ |
|  | 200 | 40.0 ± 1.5^a^ | 14.0 ± 0.5^a^ | 25.7 ± 1.0^a^ | 2 ± 1^a^ | 10 ± 2^a^ | 39.6 ± 1.9^b^ |
| **Laifeng shuiqin-1 (V11E0014)** | 0 | 52.0 ± 1.0^c^ | 19.7 ± 0.6^c^ | 32.3 ± 0.6^c^ | 3 ± 1^b^ | 27 ± 2^d^ | 33.9 ± 1.7^c^ |
|  | 50 | 45.3 ± 1.5^b^ | 18.3 ± 0.6^c^ | 27.0 ± 1.0^b^ | 4 ± 1^b^ | 22 ± 2^c^ | 34.5 ± 1.3^c^ |
|  | 100 | 40.7 ± 2.5^b^ | 13.3 ± 1.2^b^ | 27.3 ± 1.5^b^ | 3 ± 1^a^ | 17 ± 2^b^ | 29.2 ± 1.1^b^ |
|  | 200 | 39.0 ± 2.1^a^ | 12.3 ± 1.2^a^ | 25.7 ± 1.2^a^ | 3 ± 1^a^ | 12 ± 2^a^ | 24.1 ± 1.1^a^ |
| **Riben shuiqin (V11E0108)** | 0 | 53.3 ± 1.5^c^ | 19.3 ± 1.2^b^ | 34.0 ± 1.0^b^ | 5 ± 1^b^ | 34 ± 2^c^ | 31.5 ± 1.4^a^ |
|  | 50 | 46.0 ± 1.7^bc^ | 16.7 ± 1.0^ab^ | 29.0 ± 1.0^a^ | 3 ± 0^a^ | 21 ± 2^b^ | 35.4 ± 1.9^ab^ |
|  | 100 | 49.7 ± 2.9^ab^ | 19.0 ± 1.3^b^ | 30.7 ± 1.2^a^ | 3 ± 0^a^ | 17 ± 1^b^ | 36.8 ± 1.2^b^ |
|  | 200 | 44.0 ± 1.0^a^ | 15.3 ± 0.6^a^ | 28.7 ± 0.6^a^ | 3 ± 1^a^ | 13 ± 1^a^ | 35.1 ± 1.6^ab^ |
| **Wuhu gaogeng (V11E0040)** | 0 | 48.0 ± 2.0^c^ | 18.0 ± 1.0^c^ | 30.0 ± 1.7^b^ | 4 ± 1^c^ | 31 ± 2^c^ | 25.9 ± 1.4^a^ |
|  | 50 | 45.3 ± 1.5^bc^ | 14.7 ± 0.6^b^ | 30.7 ± 0.6^b^ | 4 ± 1^bc^ | 31 ± 3^c^ | 35.2 ± 1.5^c^ |
|  | 100 | 41.7 ± 1.5^ab^ | 13.7 ± 0.8^b^ | 28.0 ± 1.0^ab^ | 3 ± 0^ab^ | 20 ± 1^b^ | 38.2 ± 1.7^c^ |
|  | 200 | 38.3 ± 1.5^a^ | 11.7 ± 0.6^a^ | 26.7 ± 1.2^a^ | 2 ± 1^a^ | 15 ± 1^a^ | 30.4 ± 0.9^b^ |
| **Chenggong (V11E0031)** | 0 | 61.7 ± 1.5^b^ | 20.3 ± 0.6^b^ | 41.3 ± 1.2^b^ | 7 ± 1^b^ | 41 ± 3^b^ | 38.0 ± 2.0^a^ |
|  | 50 | 58.3 ± 1.5^ab^ | 18.0 ± 1.0^ab^ | 40.3 ± 1.2^b^ | 6 ± 1^b^ | 37 ± 1^b^ | 38.4 ± 2.1^a^ |
|  | 100 | 53.7 ± 2.1^a^ | 17.7 ± 1.5^ab^ | 36.0 ± 1.7^a^ | 4 ± 1^a^ | 26 ± 2^a^ | 39.0 ± 1.2^a^ |
|  | 200 | 52.7 ± 3.2^a^ | 17.0 ± 1.3^a^ | 35.7 ± 0.6^a^ | 4 ± 1^a^ | 23 ± 2^a^ | 36.8 ± 1.5^a^ |
| **Huaian shuiqin** | 0 | 54.7 ± 1.5^b^ | 20.7 ± 1.5^a^ | 34.0 ± 1.0^b^ | 5 ± 0^c^ | 32 ± 2^c^ | 35.8 ± 1.3^a^ |
|  | 50 | 51.7 ± 2.5^ab^ | 21.0 ± 1.0^a^ | 30.7 ± 1.5^a^ | 4 ± 0^b^ | 28 ± 2^b^ | 43.0 ± 1.9^c^ |
|  | 100 | 48.7 ± 1.5^a^ | 20.3 ± 1.5^a^ | 28.3 ± 0.6^a^ | 3 ± 0^a^ | 17 ± 1^a^ | 37.6 ± 1.8^ab^ |
|  | 200 | 47.7 ± 2.1^a^ | 19.7 ± 1.5^a^ | 28.0 ± 1.0^a^ | 3 ± 1^a^ | 15 ± 1^a^ | 41.9 ± 1.7^bc^ |
| **Hongshan gaogeng (V11E0113)** | 0 | 57.7 ± 1.2^c^ | 20.3 ± 1.5^b^ | 37.3 ± 1.5^a^ | 6 ± 0^c^ | 38 ± 1^d^ | 30.2 ± 0.8^a^ |
|  | 50 | 56.0 ± 2.0^bc^ | 21.7 ± 0.6^b^ | 34.3 ± 1.5^a^ | 5 ± 0^b^ | 31 ± 1^c^ | 32.0 ± 1.8^a^ |
|  | 100 | 51.3 ± 3.1^ab^ | 15.7 ± 1.2^a^ | 35.7 ± 2.3^a^ | 5 ± 1^b^ | 23 ± 2^b^ | 40.9 ± 1.3^c^ |
|  | 200 | 46.3 ± 1.5^a^ | 13.0 ± 1.0^a^ | 33.3 ± 1.5^a^ | 3 ± 0^a^ | 18 ± 2^a^ | 35.7 ± 1.2^b^ |
| **Qiqin**  **(V11E0175)** | 0 | 60.3 ± 2.5^b^ | 26.0 ± 2.0^c^ | 34.3 ± 0.6^b^ | 7 ± 1^b^ | 42 ± 2^c^ | 27.1 ± 1.1^a^ |
|  | 50 | 58.0 ± 1.0^ab^ | 23.0 ± 1.0^bc^ | 35.0 ± 0.0^b^ | 7 ± 1^b^ | 41 ± 1^bc^ | 33.7 ± 1.6^c^ |
|  | 100 | 53.0 ± 1.7^a^ | 21.3 ± 0.6^ab^ | 31.7 ± 1.2^a^ | 6 ± 1^ab^ | 37 ± 3^b^ | 32.1 ± 0.9^bc^ |
|  | 200 | 53.3 ± 2.9^a^ | 19.7 ± 0.6^c^ | 33.7 ± 1.5^ab^ | 5 ± 0^a^ | 29 ± 2^a^ | 30.1 ± 1.2^ab^ |
| **Shishou shuiqin (V11E0011)** | 0 | 60.0 ± 1.7^c^ | 21.7 ± 0.6^c^ | 38.3 ± 1.5^b^ | 7 ± 1^b^ | 42 ± 2^c^ | 34.4 ± 1.3^a^ |
|  | 50 | 55.0 ± 3.0^bc^ | 21.7 ± 1.5^b^ | 33.3 ± 1.5^a^ | 6 ± 1^b^ | 39 ± 2^c^ | 41.8 ± 1.7^b^ |
|  | 100 | 51.7 ± 2.1^b^ | 18.0 ± 0.9^a^ | 33.7 ± 0.6^a^ | 4 ± 1^a^ | 20 ± 2^b^ | 37.4 ± 1.4^a^ |
|  | 200 | 44.3 ± 2.3^a^ | 13.0 ± 1.0a | 31.0 ± 1.0^a^ | 3 ± 1^a^ | 16 ± 2^a^ | 37.1 ± 2.0^a^ |
| **Lujiang jiaqin (V11E0036)** | 0 | 56.0 ± 2.6^b^ | 18.7 ± 1.5^a^ | 37.3 ± 2.1^b^ | 6 ± 1^c^ | 39 ± 3^b^ | 23.2 ± 1.2^a^ |
|  | 50 | 54.7 ± 2.1^b^ | 17.0 ± 1.0^a^ | 37.7 ± 1.5^b^ | 5 ± 1^bc^ | 34 ± 2^b^ | 33.3 ± 1.0^b^ |
|  | 100 | 50.7 ± 1.5^ab^ | 18.0 ± 1.0^a^ | 32.7 ± 0.6^a^ | 4 ± 0^a^ | 21 ± 1^a^ | 35.8 ± 1.1^b^ |
|  | 200 | 46.7 ± 2.9^a^ | 17.3 ± 0.6^a^ | 29.3 ± 1.5^a^ | 4 ± 1^ab^ | 24 ± 2^a^ | 42.7 ± 1.8^c^ |
| **Tongcheng -2 (V11E0004)** | 0 | 59.3 ± 1.5^d^ | 18.3 ± 0.6^a^ | 41.0 ± 1.0^b^ | 5 ± 1^c^ | 38 ± 3^d^ | 29.9 ± 1.3^a^ |
|  | 50 | 53.0 ± 1.0^c^ | 16.7 ± 0.6^a^ | 36.3 ± 0.6^b^ | 5 ± 1^ab^ | 31 ± 3^c^ | 33.4 ± 1.0^b^ |
|  | 100 | 46.7 ± 2.3^b^ | 17.0 ± 1.3^a^ | 26.3 ± 1.5^a^ | 4 ± 1^b^ | 19 ± 2^b^ | 35.6 ± 1.2^b^ |
|  | 200 | 41.3 ± 1.5^a^ | 17.0 ± 1.0^a^ | 24.3 ± 1.2^a^ | 3 ± 1^a^ | 14 ± 1^a^ | 28.3 ± 0.9^a^ |
| **TTC shuiqin (V11E0103)** | 0 | 59.7 ± 2.1^c^ | 20.7 ± 1.2^c^ | 39.0 ± 0.0^b^ | 7 ± 1^b^ | 44 ± 2^b^ | 27.5 ± 1.1^a^ |
|  | 50 | 54.3 ± 2.1^b^ | 19.0 ± 1.0^bc^ | 38.3 ± 2.5^b^ | 7 ± 1^b^ | 40 ± 3^b^ | 39.2 ± 1.1^c^ |
|  | 100 | 47.3 ± 2.5^a^ | 15.7 ± 0.6^a^ | 31.7 ± 1.2^a^ | 4 ± 1^a^ | 20 ± 2^a^ | 32.5 ± 1.3^b^ |
|  | 200 | 45.7 ± 1.2^a^ | 17.0 ± 1.0^ab^ | 28.7 ± 0.6^a^ | 3 ± 1^a^ | 18 ± 2^a^ | 41.1 ± 1.8^c^ |
| **Mojiang putong (V11E0072)** | 0 | 56.7 ± 0.6^c^ | 18.7 ± 1.0^b^ | 38.0 ± 1.0^c^ | 6 ± 1^b^ | 40 ± 2^d^ | 30.4 ± 1.5^a^ |
|  | 50 | 52.7 ± 2.5^bc^ | 18.7 ± 1.3^b^ | 34.0 ± 1.0^b^ | 5 ± 1^b^ | 35 ± 3^c^ | 33.5 ± 1.8^a^ |
|  | 100 | 48.7 ± 1.2^ab^ | 17.0 ± 1.0^ab^ | 31.0 ± 1.7^ab^ | 3 ± 1^a^ | 24 ± 2^b^ | 39.6 ± 1.6^b^ |
|  | 200 | 45.7 ± 2.5^a^ | 15.7 ± 1.2^a^ | 30.0 ± 2.0^a^ | 3 ± 1^a^ | 15 ± 1^a^ | 34.2 ± 1.5^a^ |
| **Liuhe zhongye**  **(V11E0018)** | 0 | 57.7 ± 0.6^c^ | 19.0 ± 1.0^b^ | 38.7 ± 0.6^c^ | 5 ± 1^b^ | 36 ± 3^d^ | 26.3 ± 0.9^a^ |
|  | 50 | 53.3 ± 2.3^bc^ | 18.0 ± 1.0^b^ | 35.3 ± 1.5^bc^ | 5 ± 0^b^ | 31 ± 2^c^ | 29.7 ± 1.3^b^ |
|  | 100 | 50.3 ± 3.2^b^ | 17.7 ± 0.6^b^ | 32.7 ± 2.1^b^ | 4 ± 1^b^ | 24 ± 2^b^ | 32.3 ± 1.5^bc^ |
|  | 200 | 42.0 ± 1.7^a^ | 14.3 ± 1.0^a^ | 27.7 ± 1.2^a^ | 3 ± 1^a^ | 15 ± 1^a^ | 34.6 ± 1.3^c^ |
| **Yuqi shuiqin (V11E0003)** | 0 | 60.0 ± 1.0^b^ | 22.3 ± 1.2^c^ | 37.7 ± 1.2^b^ | 5 ± 1^b^ | 34 ± 2^d^ | 33.1 ± 1.0^a^ |
|  | 50 | 56.0 ± 1.0^b^ | 20.3 ± 0.6^bc^ | 35.7 ± 1.5^b^ | 4 ± 1^b^ | 28 ± 2^c^ | 33.8 ± 1.5^a^ |
|  | 100 | 49.7 ± 2.5^a^ | 18.3 ± 1.2^b^ | 31.3 ± 1.5^a^ | 3 ± 0^a^ | 19 ± 2^b^ | 37.8 ± 1.6^b^ |
|  | 200 | 46.0 ± 2.6^a^ | 15.7 ± 0.6^a^ | 30.3 ± 2.1^a^ | 3 ± 1^a^ | 14 ± 1^a^ | 40.0 ± 1.3^b^ |
| **Changan-1 (V11E0017)** | 0 | 66.7 ± 2.5^d^ | 20.3 ± 1.5^b^ | 45.0 ± 1.0^d^ | 6 ± 0^b^ | 44 ± 3^d^ | 31.6 ± 1.4^a^ |
|  | 50 | 58.7 ± 0.6^c^ | 20.7 ± 1.2^b^ | 38.0 ± 1.0^c^ | 5 ± 1^b^ | 34 ± 3^c^ | 34.4 ± 1.7^a^ |
|  | 100 | 49.3 ± 1.5^b^ | 16.7 ± 1.2^a^ | 32.7 ± 0.6^b^ | 3 ± 1^a^ | 20 ± 1^b^ | 41.8 ± 1.7^b^ |
|  | 200 | 43.7 ± 1.5^a^ | 15.3 ± 0.6^a^ | 28.3 ± 1.2^a^ | 3 ± 1^a^ | 14 ± 1^a^ | 32.1 ± 1.1^a^ |
| **Guangxi liuzhou**  **(V11E0050)** | 0 | 64.0 ± 2.0^c^ | 25.3 ± 1.2^b^ | 38.7 ± 1.2^b^ | 7 ± 1^c^ | 44 ± 4^d^ | 35.7 ± 1.4^a^ |
|  | 50 | 59.3 ± 1.5^bc^ | 21.3 ± 1.6^a^ | 38.0 ± 1.0^b^ | 5 ± 0^b^ | 33 ± 2^c^ | 38.6 ± 1.2^ab^ |
|  | 100 | 56.3 ± 2.3^b^ | 20.3 ± 1.5^a^ | 36.0 ± 1.0^b^ | 4 ± 0^b^ | 25 ± 1^b^ | 41.4 ± 2.0^b^ |
|  | 200 | 50.7 ± 1.2^a^ | 20.3 ± 0.6^a^ | 30.3 ± 1.5^a^ | 3 ± 1^a^ | 15 ± 1^a^ | 41.1 ± 2.2^b^ |
| **Tieshan zhongye (V11E0034)** | 0 | 66.3 ± 2.1^b^ | 20.3 ± 1.5^b^ | 46.0 ± 1.0^c^ | 6 ± 1^c^ | 40 ± 2^d^ | 33.4 ± 1.3^a^ |
|  | 50 | 61.0 ± 3.0^b^ | 22.0 ± 1.7^b^ | 39.0 ± 1.7^b^ | 6 ± 1^c^ | 34 ± 2^c^ | 38.6 ± 1.3^c^ |
|  | 100 | 52.3 ± 2.1^a^ | 16.0 ± 1.0^a^ | 36.3 ± 0.6^ab^ | 4 ± 1^b^ | 20 ± 1^b^ | 38.3 ± 1.8^bc^ |
|  | 200 | 49.0 ± 3.0^a^ | 15.0 ± 1.0^a^ | 34.0 ± 1.7^a^ | 3 ± 1^a^ | 14 ± 1^a^ | 34.6 ± 1.3^ab^ |
| **Wuhu kuanye (V11E0114)** | 0 | 56.3 ± 2.5^c^ | 20.0 ± 1.0^c^ | 36.3 ± 1.5^c^ | 6 ± 1^c^ | 40 ± 3^c^ | 34.2 ± 1.9^a^ |
|  | 50 | 48.7 ± 0.6^b^ | 17.7 ± 0.6^b^ | 31.0 ± 1.0^b^ | 5 ± 1^b^ | 28 ± 2^b^ | 32.5 ± 0.8^a^ |
|  | 100 | 42.7 ± 1.5^a^ | 15.0 ± 1.0^a^ | 27.7 ± 1.5^ab^ | 3 ± 1^ab^ | 18 ± 2^a^ | 34.1 ± 1.0^a^ |
|  | 200 | 40.7 ± 2.1^a^ | 15.7 ± 0.6^ab^ | 25.0 ± 1.0^a^ | 3 ± 1^a^ | 15 ± 1^a^ | 35.8 ± 1.4^a^ |
| **Jiangling ye (V11E0012)** | 0 | 60.3 ± 1.5^c^ | 20.7 ± 1.6^c^ | 39.7 ± 0.6^c^ | 6 ± 1^c^ | 43 ± 2^d^ | 31.8 ± 1.6^a^ |
|  | 50 | 55.3 ± 1.5^b^ | 17.7 ± 1.5^bc^ | 37.7 ± 0.6^c^ | 5 ± 1^b^ | 34 ± 2^c^ | 43.2 ± 2.0^c^ |
|  | 100 | 51.0 ± 2.6^b^ | 16.7 ± 1.2^b^ | 34.7 ± 1.2^b^ | 5 ± 1^b^ | 27 ± 2^b^ | 41.4 ± 1.4^bc^ |
|  | 200 | 43.3 ± 0.6^a^ | 12.3 ± 0.6^a^ | 31.0 ± 1.7^a^ | 3 ± 1^a^ | 20 ± 2^a^ | 37.9 ± 1.7^b^ |
| **Huaian chengxi (V11E0020)** | 0 | 55.7 ± 0.6^c^ | 16.3 ± 0.6^b^ | 39.3 ± 1.5^c^ | 6 ± 1^c^ | 42 ± 3^c^ | 33.9 ± 1.6^a^ |
|  | 50 | 46.3 ± 1.2^b^ | 14.0 ± 1.0^b^ | 32.3 ± 0.6^b^ | 5 ± 1^b^ | 30 ± 2^b^ | 42.8 ± 1.6^b^ |
|  | 100 | 44.3 ± 1.2^b^ | 14.7 ± 1.2^b^ | 29.7 ± 1.2^b^ | 4 ± 0^ab^ | 29 ± 2^b^ | 44.1 ± 2.1^b^ |
|  | 200 | 36.3 ± 1.5^a^ | 11.3 ± 0.8^a^ | 25.0 ± 1.7^a^ | 3 ± 1^a^ | 15 ± 2^a^ | 45.3 ± 2.2^b^ |
| **Xiaoqingqin (V11E0098)** | 0 | 58.7 ± 2.1^c^ | 18.0 ± 1.0^c^ | 40.7 ± 1.5^d^ | 6 ± 0^c^ | 46 ± 3^d^ | 37.9 ± 1.7^a^ |
|  | 50 | 53.7 ± 1.5^c^ | 17.3 ± 0.6^c^ | 36.3 ± 1.2^c^ | 5 ± 1^bc^ | 33 ± 2^c^ | 38.3 ± 0.9^ab^ |
|  | 100 | 45.7 ± 2.1^b^ | 14.3 ± 0.6^b^ | 31.3 ± 0.6^b^ | 5 ± 1^b^ | 27 ± 2^b^ | 41.9 ± 2.1^b^ |
|  | 200 | 40.0 ± 2.0^a^ | 12.0 ± 0.5^a^ | 28.0 ± 1.3^a^ | 3 ± 0^a^ | 15 ± 1^a^ | 35.9 ± 0.9^a^ |
| **Tongcheng (V11E0002)** | 0 | 67.3 ± 1.5^c^ | 21.0 ± 1.5^b^ | 46.3 ± 2.1^c^ | 7 ± 1^c^ | 49 ± 5^c^ | 35.5 ± 0.9^a^ |
|  | 50 | 59.3 ± 2.3^b^ | 21.0 ± 1.0^b^ | 38.3 ± 1.2^b^ | 5 ± 1^b^ | 34 ± 3^b^ | 38.1 ± 2.0^ab^ |
|  | 100 | 55.7 ± 2.5^b^ | 17.0 ± 1.0^a^ | 38.7 ± 0.6^b^ | 5 ± 0^b^ | 29 ± 2^b^ | 40.8 ± 1.5^b^ |
|  | 200 | 48.7 ± 2.5^a^ | 15.0 ± 1.0^a^ | 33.7 ± 1.5^a^ | 3 ± 1^a^ | 19 ± 1^a^ | 37.4 ± 1.6^ab^ |
| **Henei yuanye (V11E0142)** | 0 | 56.7 ± 1.5^c^ | 19.0 ± 1.0^b^ | 37.7 ± 0.6^d^ | 6 ± 1^b^ | 42 ± 3^c^ | 39.9 ± 1.6^a^ |
|  | 50 | 51.0 ± 1.0^b^ | 16.3 ± 1.3^ab^ | 35.0 ± 1.0^c^ | 5 ± 1^b^ | 32 ± 2^b^ | 45.1 ± 1.6^b^ |
|  | 100 | 46.3 ± 1.5^a^ | 15.3 ± 0.8^a^ | 31.0 ± 1.0^b^ | 4 ± 0^a^ | 29 ± 2^b^ | 45.7 ± 1.1^b^ |
|  | 200 | 42.3 ± 2.5^a^ | 15.0 ± 1.0^a^ | 27.3 ± 0.6^a^ | 4 ± 1^a^ | 23 ± 2^a^ | 43.3 ± 1.7^ab^ |
| **334 shuiqin (V11E0084)** | 0 | 63.7 ± 1.5^c^ | 20.3 ± 1.3^c^ | 43.3 ± 1.5^c^ | 6 ± 1^c^ | 47 ± 4^c^ | 36.8 ± 1.5^ab^ |
|  | 50 | 54.3 ± 2.1^b^ | 17.3 ± 1.0^b^ | 37.0 ± 1.0^b^ | 5 ± 1^bc^ | 35 ± 2^b^ | 36.1 ± 1.5^a^ |
|  | 100 | 46.7 ± 2.5^a^ | 16.3 ± 0.6^b^ | 30.3 ± 1.2^a^ | 5 ± 0^b^ | 30 ± 2^b^ | 38.1 ± 1.4^ab^ |
|  | 200 | 43.0 ± 2.6^a^ | 12.7 ± 0.6^a^ | 30.3 ± 0.6^a^ | 4 ± 1^a^ | 21 ± 2^a^ | 40.2 ± 1.1^b^ |
| **Jianshui hongqin (V11E0029)** | 0 | 60.3 ± 1.5^b^ | 22.0 ± 1.0^c^ | 38.3 ± 1.5^b^ | 6 ± 1^b^ | 46 ± 3^d^ | 37.5 ± 1.9^a^ |
|  | 50 | 56.3 ± 1.5^b^ | 20.7 ± 1.2^c^ | 35.7 ± 0.6^b^ | 6 ± 1^b^ | 35 ± 3^c^ | 39.5 ± 1.5^ab^ |
|  | 100 | 48.7 ± 2.1^a^ | 16.7 ± 1.0^cb^ | 32.0 ± 1.0^a^ | 4 ± 0^a^ | 28 ± 1^b^ | 43.5 ± 2.0^b^ |
|  | 200 | 43.7 ± 3.1^a^ | 12.7 ± 0.6^a^ | 31.0 ± 1.0^a^ | 3 ± 0^a^ | 18 ± 1^a^ | 39.6 ± 1.8^ab^ |
| **Xinsheng**  **(V11E0059)** | 0 | 62.7 ± 2.1^c^ | 25.0 ± 1.0^c^ | 37.7 ± 1.2^b^ | 6 ± 0^c^ | 42 ± 2^d^ | 32.6 ± 1.0^a^ |
|  | 50 | 57.0 ± 3.0^bc^ | 21.3 ± 1.5^b^ | 35.7 ± 0.6^b^ | 4 ± 1^b^ | 31 ± 2^c^ | 42.1 ± 1.8^b^ |
|  | 100 | 52.0 ± 1.7^ab^ | 21.3 ± 1.5^b^ | 30.7 ± 0.6^a^ | 4 ± 0^b^ | 25 ± 2^b^ | 39.3 ± 2.7^ab^ |
|  | 200 | 46.7 ± 2.5^a^ | 17.7 ± 1.2^a^ | 29.0 ± 1.7^a^ | 3 ± 0^a^ | 18 ± 2^a^ | 37.2 ± 1.3^ab^ |
| **Kunming**  **(V11E0030)** | 0 | 63.0 ± 2.0^c^ | 21.7 ± 1.5^b^ | 41.3 ± 0.6^c^ | 7 ± 0^d^ | 49 ± 2^c^ | 35.1 ± 1.6^a^ |
|  | 50 | 55.0 ± 2.0^b^ | 19.7 ± 1.5^b^ | 35.3 ± 0.6^b^ | 5 ± 1^c^ | 34 ± 2^b^ | 35.6 ± 1.5^a^ |
|  | 100 | 53.3 ± 2.5^b^ | 21.3 ± 1.5^b^ | 32.0 ± 1.0^a^ | 4 ± 0^b^ | 32 ± 2^b^ | 43.0 ± 2.0^b^ |
|  | 200 | 45.3 ± 2.5^a^ | 13.7 ± 1.3^a^ | 31.7 ± 0.6^a^ | 3 ± 0^a^ | 19 ± 2^a^ | 33.7 ± 1.4^a^ |
| **Qufu**  **(V11E0053)** | 0 | 60.7 ± 2.1^c^ | 22.7 ± 1.5^b^ | 38.0 ± 1.0^c^ | 6 ± 1^c^ | 40 ± 3^c^ | 31.6 ± 1.3^a^ |
|  | 50 | 56.3 ± 2.1^bc^ | 21.7 ± 1.2^b^ | 34.7 ± 1.2^b^ | 5 ± 1^bc^ | 32 ± 3^b^ | 38.1 ± 1.8^b^ |
|  | 100 | 52.0 ± 2.6^b^ | 19.7 ± 1.5^ab^ | 32.3 ± 1.2^b^ | 4 ± 1^b^ | 30 ± 2^b^ | 43.4 ± 1.2^c^ |
|  | 200 | 45.0 ± 2.0 ^a^ | 16.3 ± 1.0^a^ | 28.7 ± 1.5^a^ | 3 ± 1^a^ | 20 ± 2^a^ | 42.3 ± 1.7^c^ |
| **Renhezhen-2 (V11E0123)** | 0 | 63.3 ± 1.5^d^ | 21.7 ± 1.2^c^ | 41.3 ± 0.6^c^ | 8 ± 1^d^ | 56 ± 3^d^ | 30.6 ± 1.3^a^ |
|  | 50 | 57.7 ± 1.5^c^ | 20.0 ± 1.7^bc^ | 37.7 ± 0.6^b^ | 6 ± 0^c^ | 40 ± 2^c^ | 31.6 ± 1.3^a^ |
|  | 100 | 49.7 ± 2.1^b^ | 17.3 ± 0.6^b^ | 32.3 ± 1.5^a^ | 4 ± 0^b^ | 29 ± 2^b^ | 31.7 ± 1.4^a^ |
|  | 200 | 44.0 ± 1.0^a^ | 13.3 ± 1.2^a^ | 30.7 ± 1.2^a^ | 3 ± 0^a^ | 21 ± 2^a^ | 32.9 ± 1.4^a^ |
| **Guanyin dongye (V11E0145)** | 0 | 54.0 ± 1.0^d^ | 21.3 ± 1.5^c^ | 32.7 ± 1.2^c^ | 6 ± 1^c^ | 44 ± 3^d^ | 33.2 ± 1.6^a^ |
|  | 50 | 48.3 ± 1.5^c^ | 19.7 ± 0.6^c^ | 28.7 ± 1.2^b^ | 6 ± 1^c^ | 35 ± 2^c^ | 34.4 ± 1.6^ab^ |
|  | 100 | 43.0 ± 1.0^b^ | 17.3 ± 1.2^b^ | 25.7 ± 0.6^a^ | 4 ± 0^b^ | 27 ± 2^b^ | 39.3 ± 1.8^c^ |
|  | 200 | 39.7 ± 1.5^a^ | 15.0 ± 1.0^a^ | 24.7 ± 0.6^a^ | 3 ± 1^a^ | 21 ± 1^a^ | 37.4 ± 1.2^bc^ |
| **Chenggong-2 (V11E0096)** | 0 | 65.0 ± 2.0^c^ | 21.7 ± 0.6^c^ | 43.3 ± 1.5^d^ | 7 ± 1^c^ | 53 ± 4^c^ | 34.4 ± 1.6^a^ |
|  | 50 | 58.0 ± 2.0^b^ | 20.3 ± 1.2^bc^ | 37.7 ± 0.6^c^ | 5 ± 1^c^ | 35 ± 2^b^ | 33.2 ± 1.7^a^ |
|  | 100 | 53.0 ± 2.0^b^ | 18.7 ± 1.2^b^ | 34.3 ± 1.2^b^ | 5 ± 1^b^ | 31 ± 2^b^ | 42.7 ± 1.5^b^ |
|  | 200 | 45.0 ± 1.7^a^ | 14.7 ± 1.2^a^ | 30.3 ± 0.6^a^ | 4 ± 1^a^ | 21 ± 2^a^ | 42.2 ± 2.0^b^ |
| **Emei shan-2 (V11E0125)** | 0 | 56.3 ± 1.5^c^ | 19.3 ± 1.2^b^ | 37.0 ± 1.0^c^ | 6 ± 1^c^ | 48 ± 3^d^ | 31.3 ± 1.0^a^ |
|  | 50 | 49.0 ± 1.7^b^ | 17.7 ± 1.2^b^ | 31.3 ± 0.6^b^ | 5 ± 1^b^ | 37 ± 3^c^ | 41.1 ± 1.9^b^ |
|  | 100 | 42.7 ± 1.2^a^ | 14.3 ± 1.0^a^ | 28.3 ± 1.5^ab^ | 4 ± 1^a^ | 29 ± 2^b^ | 39.0 ± 1.5^b^ |
|  | 200 | 40.0 ± 2.6^a^ | 13.3 ± 1.0^a^ | 26.7 ± 1.5^a^ | 4 ± 0^a^ | 20 ± 2^a^ | 38.7 ± 1.4^b^ |
| **Hongshan Lv geng (V11E0044)** | 0 | 61.7 ± 2.5^c^ | 20.7 ± 1.5^b^ | 41.0 ± 1.0^d^ | 7 ± 1^c^ | 48 ± 3^c^ | 30.7 ± 1.6^a^ |
|  | 50 | 57.0 ± 1.7^bc^ | 19.0 ± 1.0^ab^ | 38.0 ± 1.0^c^ | 6 ± 1^bc^ | 37 ± 2^b^ | 39.6 ± 1.0^b^ |
|  | 100 | 51.3 ± 2.5^b^ | 17.0 ± 1.5^a^ | 34.3 ± 0.6^b^ | 5 ± 0^b^ | 34 ± 1^b^ | 44.9 ± 1.4^c^ |
|  | 200 | 44.7 ± 2.1^a^ | 15.7 ± 1.2^a^ | 29.0 ± 1.0^a^ | 3 ± 0^a^ | 18 ± 1^a^ | 42.9 ± 1.6^bc^ |
| **Anhui danyang (V11E0129)** | 0 | 64.0 ± 1.0^c^ | 21.0 ± 1.0^b^ | 43.0 ± 1.0^d^ | 7 ± 1^b^ | 55 ± 4^d^ | 27.8 ± 0.9^a^ |
|  | 50 | 56.3 ± 2.1^b^ | 18.7 ± 1.5^b^ | 37.7 ± 0.6^c^ | 6 ± 1^b^ | 43 ± 3^c^ | 33.2 ± 1.2^b^ |
|  | 100 | 46.3 ± 2.3^a^ | 15.0 ± 1.0^a^ | 31.3 ± 0.6^b^ | 4 ± 0^b^ | 26 ± 1^b^ | 36.8 ± 1.7^b^ |
|  | 200 | 41.7 ± 2.1^a^ | 13.3 ± 1.0^a^ | 28.3 ± 0.6^a^ | 3 ± 0^a^ | 17 ± 1^a^ | 36.7 ± 1.7^b^ |
| **Changshu shuiqin** | 0 | 63.3 ± 2.5^d^ | 22.0 ± 1.7^c^ | 41.3 ± 0.6^d^ | 6 ± 0^c^ | 50 ± 3^d^ | 35.9 ± 1.7^a^ |
|  | 50 | 57.7 ± 0.6^c^ | 20.0 ± 1.0^bc^ | 36.3 ± 1.5^c^ | 5 ± 1^bc^ | 36 ± 2^c^ | 38.0 ± 1.2^ab^ |
|  | 100 | 51.7 ± 1.5^b^ | 18.3 ± 1.2^b^ | 33.3 ± 0.6^b^ | 4 ± 1^b^ | 28 ± 2^b^ | 40.4 ± 1.7^b^ |
|  | 200 | 45.3 ± 1.2^a^ | 15.0 ± 1.0^a^ | 30.3 ± 0.6^a^ | 3 ± 1^a^ | 17 ± 1^a^ | 39.8 ± 1.7^ab^ |
| **Shaoyang ye (V11E0094)** | 0 | 61.7 ± 1.5^c^ | 20.3 ± 1.5^c^ | 41.3 ± 1.5^c^ | 7 ± 1^b^ | 59 ± 3^d^ | 32.1 ± 1.4^a^ |
|  | 50 | 53.3 ± 2.1^b^ | 16.0 ± 1.0^b^ | 37.3 ± 1.5^b^ | 6 ± 1^b^ | 41 ± 3^c^ | 36.1 ± 1.5^bc^ |
|  | 100 | 46.7 ± 2.5^a^ | 15.0 ± 1.0^ab^ | 31.3 ± 1.2^a^ | 5 ± 1^a^ | 31 ± 2^b^ | 38.6 ± 1.4^c^ |
|  | 200 | 42.3 ± 2.5^a^ | 12.3 ± 1.0^a^ | 30.0 ± 0.0^a^ | 4 ± 0^a^ | 24 ± 1^a^ | 34.3 ± 1.3^ab^ |
| **Jinjipo**  **(V11E0009)** | 0 | 61.7 ± 0.6^c^ | 16.7 ± 1.2^c^ | 45.0 ± 1.7^c^ | 6 ± 1^b^ | 49 ± 4^c^ | 32.8 ± 1.6^a^ |
|  | 50 | 55.7 ± 1.2^b^ | 15.7 ± 1.2^bc^ | 40.0 ± 1.0^b^ | 5 ± 1^b^ | 39 ± 2^b^ | 34.0 ± 1.4^a^ |
|  | 100 | 48.0 ± 2.0^a^ | 13.7 ± 1.2^ab^ | 34.3 ± 1.5^a^ | 4 ± 0^a^ | 30 ± 2^a^ | 39.4 ± 1.8^b^ |
|  | 200 | 46.3 ± 2.5^a^ | 12.7 ± 0.3^a^ | 33.7 ± 1.2^a^ | 4 ± 0^a^ | 24 ± 1^a^ | 35.2 ± 1.6^a^ |
| **Suzhou**  **(V11E0137)** | 0 | 64.7 ± 2.1^b^ | 21.0 ± 1.7^b^ | 43.7 ± 1.5^c^ | 6 ± 0^c^ | 42 ± 2^c^ | 35.5 ± 1.4^a^ |
|  | 50 | 60.0 ± 3.0^b^ | 20.0 ± 1.7^b^ | 40.0 ± 1.7^bc^ | 5 ± 1^c^ | 34 ± 3^b^ | 36.1 ± 1.4^a^ |
|  | 100 | 50.3 ± 2.1^a^ | 12.7 ± 0.6^a^ | 37.7 ± 1.5^b^ | 4 ± 0^b^ | 30 ± 2^b^ | 41.4 ± 1.0^b^ |
|  | 200 | 47.3 ± 2.5^a^ | 15.7 ± 1.2^a^ | 31.7 ± 0.6^a^ | 3 ± 0^a^ | 21 ± 2^a^ | 42.7 ± 1.3^b^ |
